# Cyclophosphamide depletes tumor infiltrating T regulatory cells and combined with anti-PD-1 therapy improves survival in murine neuroblastoma

**DOI:** 10.1016/j.isci.2022.104995

**Published:** 2022-08-23

**Authors:** Emily R. Webb, Julia Moreno-Vincente, Alistair Easton, Silvia Lanati, Martin Taylor, Sonya James, Emily L. Williams, Vikki English, Chris Penfold, Stephen A. Beers, Juliet C. Gray

**Affiliations:** 1 Antibody and Vaccine Group, Centre for Cancer Immunology, University of Southampton Faculty of Medicine, Tremona Road, Southampton, Hampshire SO16 6YD, UK; 2 Cellular Pathology, University Hospitals Southampton NHS Foundation Trust, Southampton SO16 6YD, UK

**Keywords:** Microenvironment, Biological sciences, Immunology, Cancer

## Abstract

The outcome for children with high-risk neuroblastoma is poor despite intensive multi-modal treatment protocols. Toxicity from current treatments is significant, and novel approaches are needed to improve outcome. Cyclophosphamide (CPM) is a key component of current chemotherapy regimens and is known to have immunomodulatory effects. However, this has not been investigated in the context of tumor infiltrating lymphocytes in neuroblastoma. Using murine models of neuroblastoma, the immunomodulatory effects of low-dose CPM were investigated using detailed immunophenotyping. We demonstrated that CPM resulted in a specific depletion of intratumoral T regulatory cells by apoptosis, and when combined with anti-PD-1 antibody therapy, this resulted in improved therapeutic efficacy. CPM combined with anti-PD-1 therapy was demonstrated to be an effective combinational therapy, with metronomic CPM found to be more effective than single dosing in more resistant tumor models. Overall, this pre-clinical data strongly support clinical evaluation of such combination strategies in neuroblastoma.

## Highlights

• Low dose cyclophosphamide selectively depletes tumor Tregs in neuroblastoma models
• Depletion of tumor Tregs is transient and dependent on apoptosis
• Cyclophosphamide and anti-PD-1 mAb are an effective combinational therapy
• Metronomic cyclophosphamide is more effective than a single dose in resistant tumors

## Graphical abstract

**Figure:**
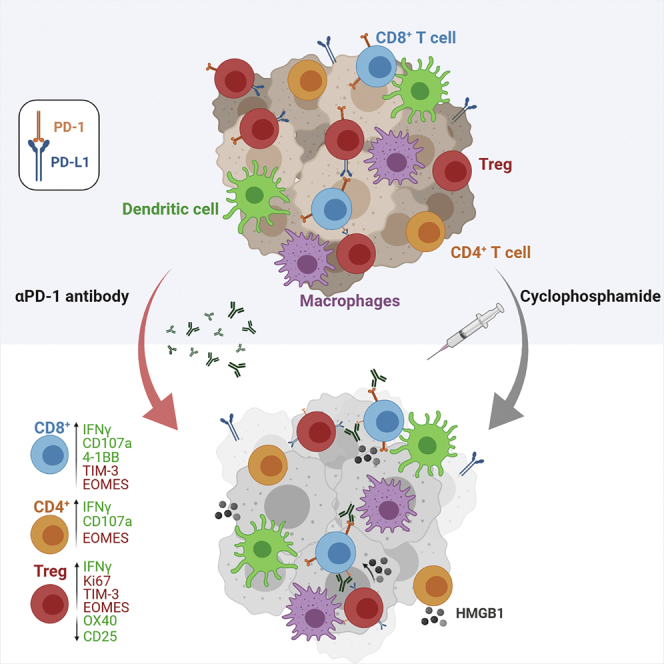

## Introduction

Neuroblastoma, an embryonal tumor derived from neural crest cells, is one of the most common pediatric malignancies and accounts for a disproportionately high number of childhood cancer deaths (Cheung and Dyer, 2013; Coughlan et al., 2017; Maris et al., 2007; Matthay et al., 2016). Over 50% of patients have evidence of MYCN amplification and/or metastatic disease and are therefore considered to have high-risk neuroblastoma (Cheung and Dyer, 2013; Coughlan et al., 2017; Maris et al., 2007; Matthay et al., 2016). Despite intensive multi-modal treatment protocols, including myeloablative chemotherapy and autologous stem cell transplant, the outcome for these patients is poor, with long-term survival rates of less than 50% (Cheung and Dyer, 2013; Coughlan et al., 2017; Maris et al., 2007; Matthay et al., 2016). Current treatment protocols are associated with significant toxicity and treatment related mortality, so there is little scope for further intensification (Pinto et al., 2015; Yu et al., 2010). There is therefore a need for new treatment strategies to reduce toxicity and improve outcome.

In the last decade, immunotherapy has become a key component of high-risk neuroblastoma treatment. Antibodies targeting the GD2 ganglioside, expressed ubiquitously on neuroblastoma, have been demonstrated to improve survival, and are now considered a standard of care (Ladenstein et al., 2018; Yu et al., 2010). However, trials of checkpoint blockade immunotherapy have so far been disappointing, with single agent anti-PD-1 antibody therapy showing little evidence of efficacy in pediatric cancers, including neuroblastoma (Davis et al., 2020; Geoerger et al., 2020). This is perhaps not surprising, given that these tumors generally have a low mutational burden and an immunologically cold microenvironment, with few infiltrating lymphocytes (Carlson et al., 2013; Coughlin et al., 2006; Camisaschi et al., 2018; Gröbner et al., 2018). Combinational approaches have been widely explored as a strategy to improve the efficacy of anti-PD-1 antibodies, although studies investigating this in the context of neuroblastoma are limited. Although initially thought to be counterintuitive, many pre-clinical and clinical studies have demonstrated benefit from combining checkpoint blockade inhibitors with chemotherapy. It is now well-recognized that many conventional chemotherapy agents may have immunomodulatory effects which may be beneficial in terms of generating an anti-tumor immune response. A number of chemotherapies, including doxorubicin and cyclophosphamide (CPM), have been proposed to induce ‘immunogenic cell death’ (ICD) of tumor cells, characterized by release or ectopic exposure of particular molecules which in turn prime an immune response. Factors involved include the cell surface expression of calreticulin (ecto-CRT) and heat-shock protein-70 (HSP-70), and the release of adenosine triphosphate (ATP) and high-mobility group box 1 (HMGB1) (Ahmed and Tait, 2020; Sistigu et al., 2014). ICD has been reported to be initiated by specific chemotherapies, radiotherapy and some viruses, and has been investigated in numerous cancer models (Vanmeerbeek et al., 2020; Ahmed and Tait, 2020). In addition to these potential effects of chemotherapy on tumor cells, a number of agents have also been reported to have favorable effects on the tumor immune microenvironment. For instance, CPM has been demonstrated to deplete regulatory T cells (Treg) in a range of cancer types (Buccione et al., 2018; Burlion et al., 2019; Du and Waxman, 2020; Guillerey et al., 2019; Loyher et al., 2016; Scurr et al., 2017).

Here we have investigated combining chemotherapy with an anti-PD-1 monoclonal antibody in pre-clinical neuroblastoma models. We chose to focus on CPM, because it is already widely used in the upfront and relapse treatment of neuroblastoma and has the potential to induce ICD as well as to modulate to immune microenvironment through Treg depletion. Because mice metabolize CPM differently to humans, they are less sensitive to the drug. Therefore, doses used here are not equated directly to doses used in the clinic, but are still considered ‘low dose’ within a murine context (Ramirez et al., 2019).

Here, we have demonstrated that a relatively low dose of CPM led to depletion of Treg specifically within the tumor microenvironment, and phenotypic modulation of other T cell subsets. Combination with anti-PD-1 antibody therapy resulted in an increase in survival in pre-clinical models. However, the Treg depletion was observed to be transient, therefore metronomic dosing of both chemotherapy and antibody therapy led to improvement in survival in more resistant pre-clinical models of neuroblastoma.

## Results

### Cyclophosphamide induces upregulation of markers of immunogenic cell death in murine neuroblastoma

**Figure 1:**
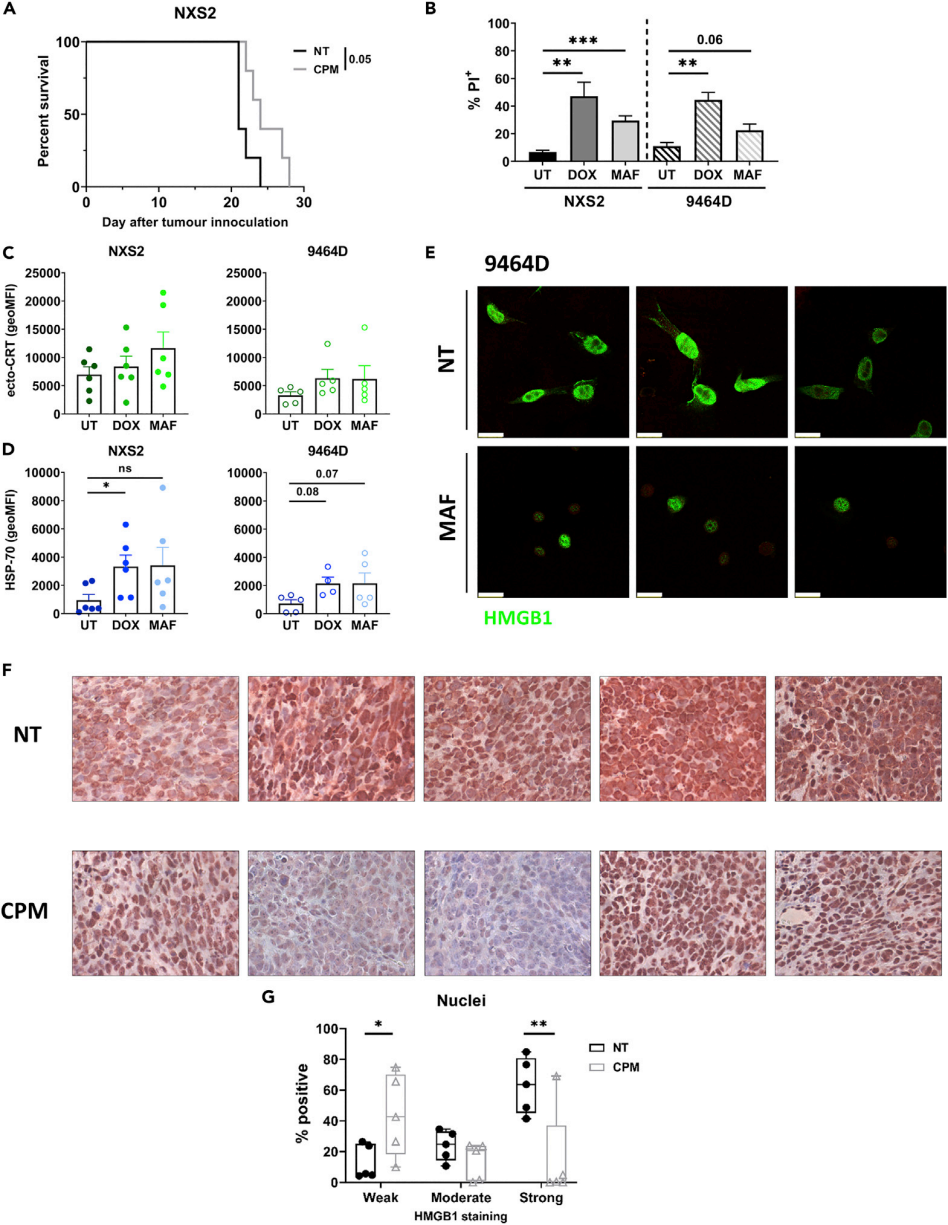
Murine neuroblastoma cell lines are susceptible to immunogenic cell death induction after chemotherapy application *in vitro* (A) NXS2 subcutaneous tumor bearing mice were treated with 40 mg/kg of CPM i.p. once tumors reached approximately 8 × 8 mm. Tumors were measured until endpoint was reached, and a survival curve generated. n = 5 per group. Examples shown of at least two separate experiments. (B–D) NXS2 or 9464D cells were treated with either DOX (40 μM), MAF (NXS2 = 50 μg/mL; 9464D = 75 μg/mL) or DMSO control (UT) for 24 h. Cells were then assessed for PI expression which denotes cell death C) ecto-CRT or D) HSP-70 by flow cytometry, using gating shown in Figure S1B. (E) Immunofluorecent staining of HMGB1 in 9464D cells 24 h after MAF treatment. Scale bar= 100 μm. Data collected over 4 separate experiments (A-E). (F) FFPE 9464D tumors Day 3 after CPM (40 mg/kg) or PBS i.p., were stained for HMGB1 expression. n= 5 mice per group, from one experiment. 10x magnification. (G) Quantification of HMGB1 staining of nuclei of cells stained in (F). Data are represented as mean ±SD Significance was assessed by LogRank test (A) or t test (B–D and G) with ∗ = p <0.05, ∗∗ = p<0.01 and ∗∗∗ = p<0.01. See also Figures S1 and S2.

CPM has been previously reported to induce ICD in the context of a range of tumor types (Vanmeerbeek et al., 2020; Du and Waxman, 2020; Gebremeskel et al., 2017; Rossi et al., 2020). Therefore, we investigated the effects of this agent in murine models of neuroblastoma. Firstly, it was observed that administration of a single low-dose of CPM (40 mg/kg), led to an increase in survival compared to non-treated (NT) mice in the immunogenic NXS2 subcutaneous neuroblastoma model (Figure 1A). To establish if ICD mechanisms potentially contribute to this survival benefit, we investigated the effects of mafosfamide (MAF), the active metabolite of CPM, in two cell lines, and compared with the effects of doxorubicin (DOX), which has been widely reported to induce ICD (Vanmeerbeek et al., 2020; Galluzzi et al., 2012; Casares et al., 2005). Viability assays were conducted to establish cytotoxic doses of the drugs in each cell line (Figure S1A and S1B), which were used in subsequent experiments to look for expression of markers of ICD. Both MAF and DOX demonstrated significant cytotoxicity to the neuroblastoma cell lines (Figure 1B). Expression levels of cell surface calreticulin (ecto-CRT) and HSP-70 were determined using flow cytometry (Figures 1C, 1D, and S1C). Exposure to the agents resulted in a significant increase in HSP-70 expression in both cell lines. However, significant changes in ecto-CRT were not observed. Finally, HMGB1 expression was assessed both *in vitro* and *in vivo*. It has been reported that after ICD induction, nuclear HMGB1 can translocate to the cytoplasm and be released into the extracellular space (Ahmed and Tait, 2020; Apetoh et al., 2007). Here, we demonstrated that MAF treatment of 9464D cells led to changes in cellular distribution of HMGB1 compared to untreated cells (Figure 1E), with clear changes in cell morphology. Furthermore, changes in the distribution of HMGB1 were observed when 9464D tumor bearing mice were administered CPM, with a decrease in strong nuclear staining compared to untreated tumors (Figures 1F, 1G, and S2). Overall, these *in vitro* and *in vivo* data suggest that CPM and DOX may lead to the induction of some features ascribed to ICD in NB models.

### Low-dose cyclophosphamide results in depletion of tumor infiltrating Tregs

**Figure 2:**
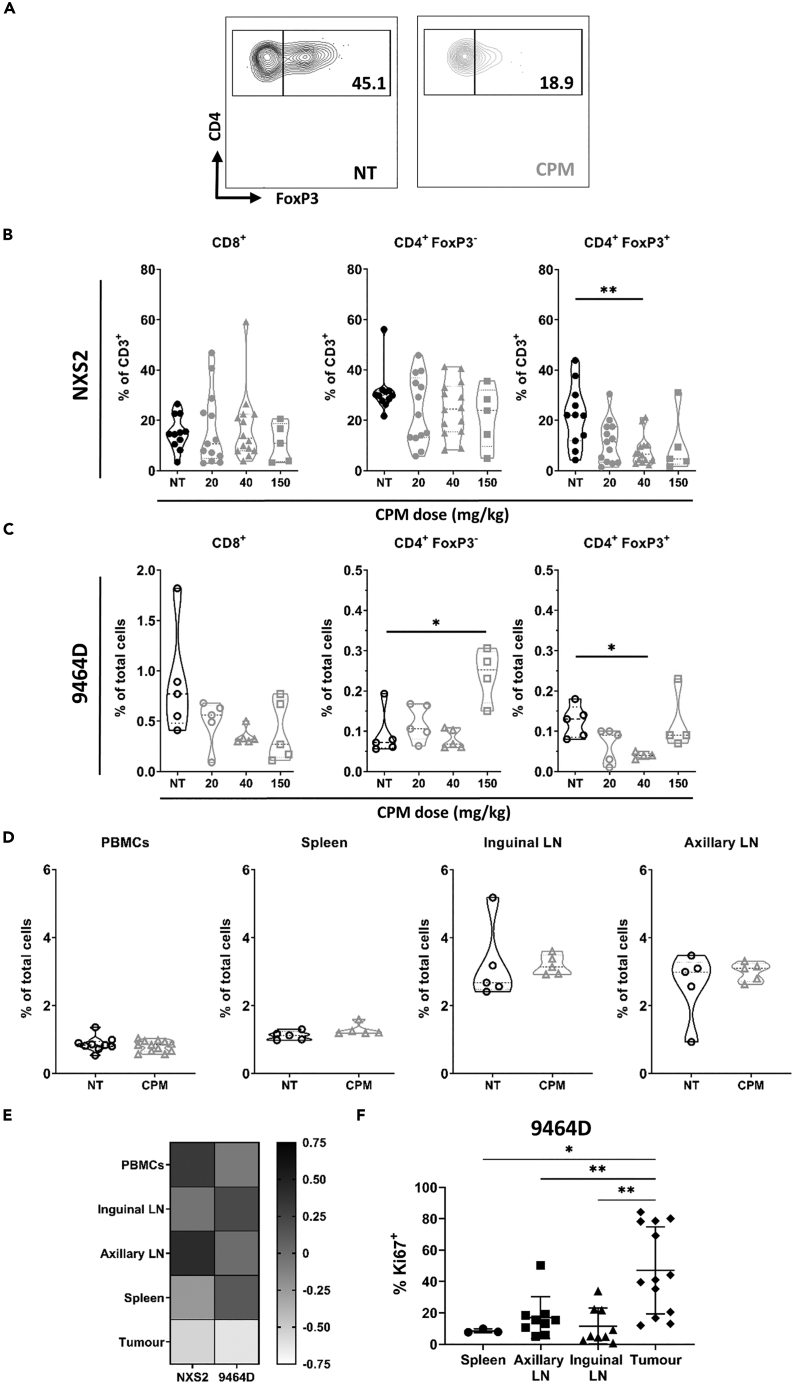
Low dose cyclophosphamide leads to selective depletion of intratumoral Treg cells in neuroblastoma models (A–C)Mice bearing either NXS2 (A + B) or 9464D (C) tumors were treated with CPM i.p. and tissues harvested at day 3 for immunophenotyping by flow cytometry (Fig.S3A-C). Contour plot example of CD4^+^ FoxP3^+^ populations as quantified in A + B, with percentage of CD4^+^ cells shown (A). Proportion of cells are shown as either % of CD3^+^ cells (B) or % of total cells (C) Combination of two experiments, with n = 11 (NT); 13 (20); 14 (40); 5 (150) for NXS2 (A + B) and n = 5 for 9464D (C). (D) Percentage of CD4^+^ FoxP3^+^ cells as % of total cells for 9464D tumor bearing mice, treated as described for (A–C). Combination of two experiments with n= 9–12 (PBMCs); n = 5 (spleen and lymph nodes). (E) Heatmap demonstrating the fold change of CD4^+^ FoxP3^+^ cell percentage of the CPM treated tissue over the NT tissue for both NXS2 and 9464D tumor bearing mice. (F) Percentage Ki67^+^ cells shown as a percentage of FoxP3^+^ cells in numerous tissues of 9464D tumor bearing mice. N = 3 (spleen); n= 9 (Axillary and inguinal LN); n = 13 (tumor). Performed over at least 2 experiments. LN, lymph node. Data are represented as mean ±SD Significance was assessed by t-test with ∗ = p <0.05, ∗∗ = p <0.01. See also Figures S3–S6.

In addition to possible ICD inducing properties, it has previously been reported that CPM can directly modify the tumor microenvironment, with modulation of intratumoral T cell numbers observed in several different cancers (Scurr et al., 2017; Buccione et al., 2018; Burlion et al., 2019; Du and Waxman, 2020; Guillerey et al., 2019; Loyher et al., 2016). However, the majority of these pre-clinical studies used relatively high doses of CPM, and the effects of low-dose CPM (<100 mg/kg) are less clear. To this end, NXS2 and 9464D tumor bearing mice were treated with CPM and after 72 h, the tumors harvested for analysis of immune cell populations by flow cytometry. For both models, no significant alterations in the percentage of effector T cells (CD8^+^ and CD4^+^ FoxP3^-^) were demonstrated, except at the highest doses of CPM (Figures 2A and 2B). However, the percentage of Treg (CD4^+^ FoxP3^+^) cells was significantly reduced in both models, even at a low dose of CPM (40 mg/kg) (Figures 2A–2C and S4A). In comparison, administration of DOX to NSX2 tumor bearing mice did not result in any significant reduction in the number of Tregs (Figure S4B). Administration of low dose CPM did not have any significant systemic effects on the percentage of T cell populations in both models (Figures 2D, 2E, S4C, and S4D). Furthermore, increased Ki67 expression was noted in intratumoral Tregs in non-treated mice of both models, as compared to non-tumor tissues, suggesting these cells have higher levels of proliferation (Figures 2F and S4E). As CPM targets proliferating cells (Voelcker, 2020), this could support the contention that tumor infiltrating Tregs may be more sensitive to CPM, and may explain the tumor specific deletion observed. Minimal modulation of the percentage of other immune subsets, including myeloid populations, NK, NKT, and B cells, was observed at lower doses of CPM (<40 mg/kg), with significant changes demonstrated only at a high dose of 150 mg/kg (Figure S5). Although the percentage of myeloid cells was not affected, changes in FcγR expression did occur (Figure S6). The FcγR expression profile reflects the phenotype of myeloid cells, and importantly can impact both positively and negatively on antibody effector functions, depending on which FcγR the antibody binds to (Nimmerjahn et al., 2015). Here, an increase of FcγRI was observed on most of the myeloid cell types analyzed (Figure S6A), together with an increase in the activating:inhibitory (A:I) ratio on monocytes and dendritic cells at Day 3 after CPM (Figure S6B), suggesting activation of these cells. In summary, Treg depletion within the TME was achieved with a relatively low dose of CPM (40 mg/kg), without overt changes in the numbers of infiltrating effector T cells. Therefore the 40 mg/kg dose, which was demonstrated in Figures 1A to a have therapeutic effect, was used as a ‘low-dose’ for further investigation into CPM immunomodulatory capacity.

### Low-dose cyclophosphamide results in modulation of tumor infiltrating T cell phenotypes

**Figure 3:**
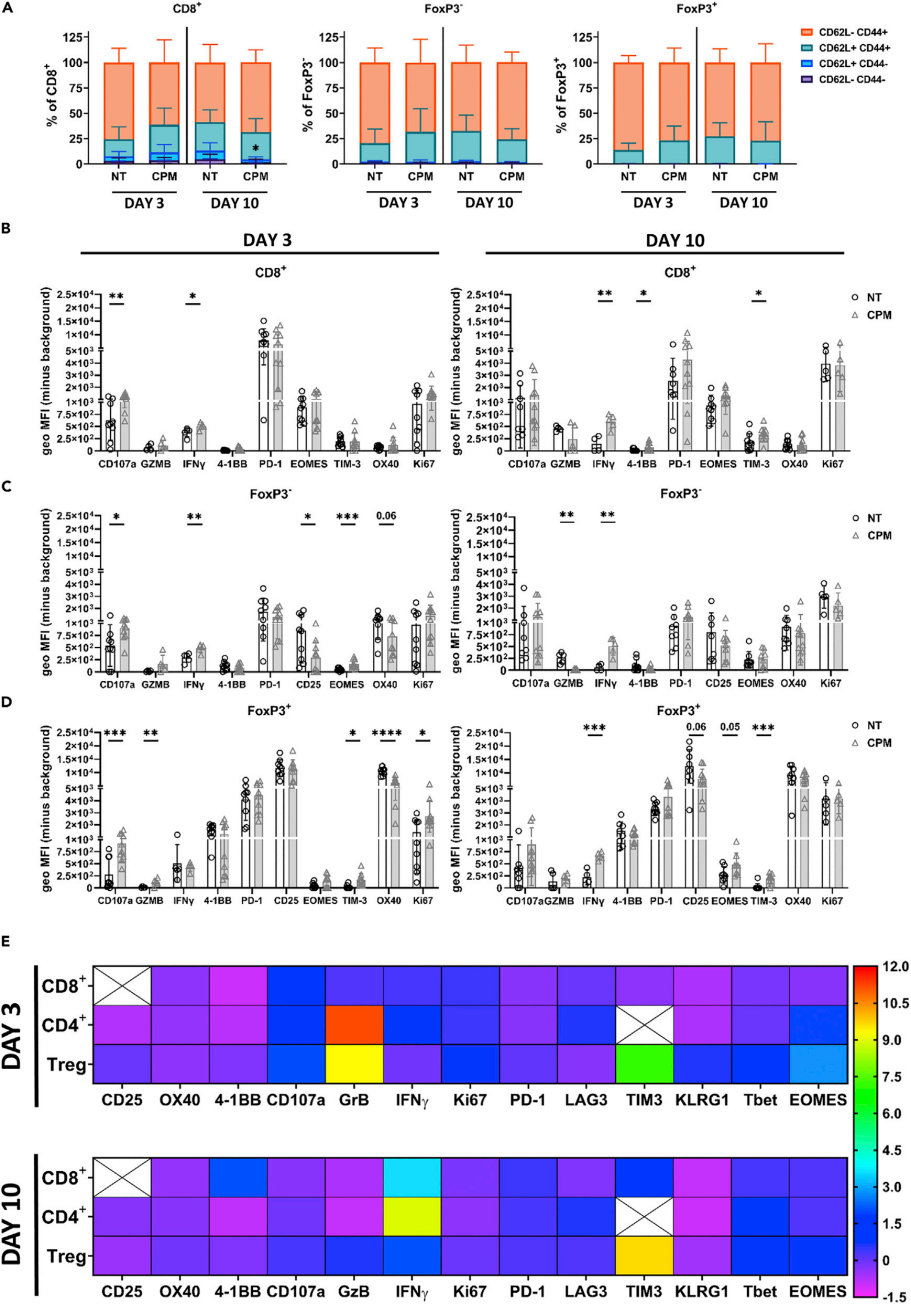
Low dose cyclophosphamide leads to modulation of T cell phenotypes in TILs of subcutaneous neuroblastoma models (A–D)Mice bearing 9464D tumors were injected with 40 mg/kg CPM i.p and tissues harvested at day 3 for immunophenotyping analysis, using flow cytometry (Fig.S3B-D). Percentage of memory populations of CD8^+^, CD4^+^ and Treg cells were determined. N = 10 per group, performed over two experiments B-D) geoMFI expression of several T cell proteins was assessed at both Day 3 and Day 10, on CD8^+^ (B), FoxP3^-^ (C) and FoxP3^+^ (D). (E) Summary heatmap of fold change over the mean of NT of all proteins assessed by flow cytometry on CD8^+^, CD4^+^ and Treg cells at both Day 3 and 10 after CPM. n= 4–10, performed over 2 experiments. Data are represented as mean ±SD Significance was assessed by t-test with ∗ = p <0.05, ∗∗ = p <0.01, ∗∗∗ = p <0.001, ∗∗∗∗ = p<0.0001. See also Figures S3D and S7.

Having demonstrated a specific depletion of Tregs within the TME, further immunophenotyping was conducted to examine the effects of low dose CPM on other T cell populations (Figures 3 and S3). First, a reduction in the proportion of infiltrating naive CD8^+^ (CD62L^+^ CD44^−^) T cells was observed on Day 10 after CPM (Figure 3A), with no other changes in expression pattern of memory associated markers. Expression of established T cell phenotypic markers were also studied (Figures 3B–3D and S7), and summarized in Figure 3E. CD8^+^ cells demonstrated an increase in CD107a expression at Day 3, however this difference was lost at Day 10 (Figure 3B). Furthermore, at Day 10 a significant increase in the proportion of 4-1BB, IFNγ and TIM-3 expressing cells was noted (Figures 3B and S7A), suggesting CD8^+^ cells in CPM tumors were initially activated, before changing to a more ‘exhausted’ phenotype in CPM treated tumors. CD4^+^ FoxP3^-^ cells also demonstrated increased CD107a expression at Day 3 after CPM, alongside an increased proportion of 4-1BB^+^ cells (Figures 3C and S7B). By Day 10, a significant increase in IFNγ expression was observed, alongside an increased proportion of EOMES expressing cells. Together, these data demonstrate that CPM leads to an initial activation of CD4^+^ FoxP3^-^ cells, resulting in expression of ‘exhaustion’ markers at later time-points. Finally, Treg (FoxP3^+^) cells again demonstrated an increase in CD107a^+^ cells at Day 3, alongside an increase in TIM-3 and Ki67 expression (Figures 3D and S7C). Furthermore, a reduction in CD25 and OX40 expression at Day 3 after CPM was also observed, which was still decreased at Day 10 for CD25. Finally, a significant increase in TIM-3 was also seen at Day 10, with an increase in EOMES. Changes in CD25 and OX40 expression demonstrated here, could suggest that the remaining Tregs have reduced functionality after CPM (Willoughby et al., 2017; Griseri et al., 2010; Plitas and Rudensky, 2016). A summary heatmap of all phenotyping data is shown in Figure 3E. Overall, low-dose CPM was demonstrated to modulate the phenotypes of 3 different T cell subsets, with significant effects on Treg phenotype.

### Low-dose cyclophosphamide induced Treg depletion and therapy is abrogated in apoptosis resistant mice

**Figure 4:**
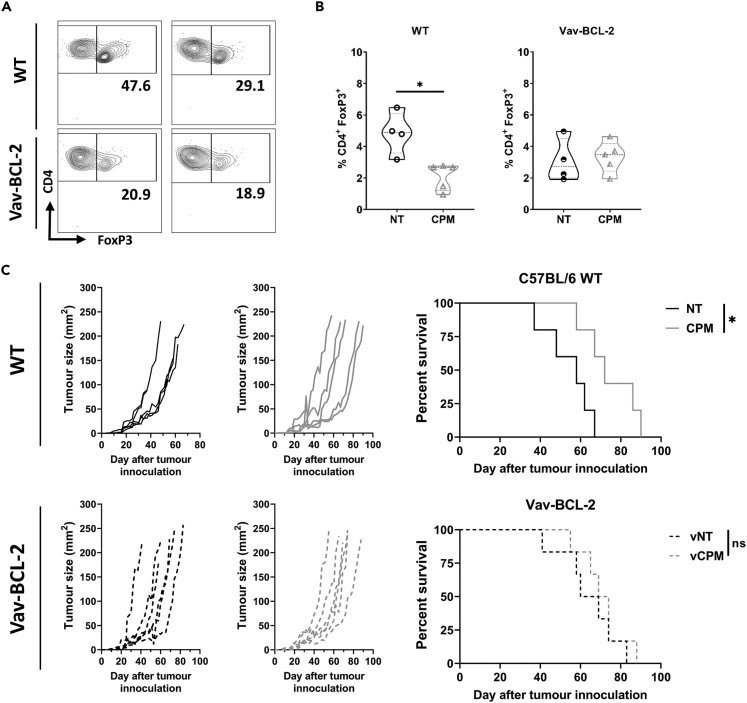
Overexpression of BCL-2 in Tregs prevents depletion by CPM and ablates efficacy of combination therapy *in vivo* (A and B) WildType (WT) C57BL/6 mice or vav-BCL-2 mice were injected subcutaneously with 9464D cells, then treated with 40 mg/kg CPM i.p. and tumors harvested at day 3 for immunophenotyping by flow cytometry (gating strategy shown in Fig.S3B-D). Examples of contour plots of FoxP3 and CD4 expression is shown on the left, with B) quantification of the percentage of Treg cells demonstrated on the right. n= 5 (CPM) n= 4 (NT). (C) Tumor growth (left) and survival (right) of 9464D tumor bearing WT or vav-BCL-2 mice after single treatment with 40 mg/kg CPM i.p at 5 × 5 mm tumor size. n= 5–6 per group. Combined data from two independent experiments (A–C). Data are represented as mean ±SD Significance was assessed by either t-test (B) or Log-Rank test (C) with ∗ = p <0.05.

As demonstrated above (Figures 2 and 3), low-dose CPM was able to significantly deplete and modulate Treg cells within the neuroblastoma TME. To elucidate whether Treg depletion was important to the therapeutic effectiveness of CPM, vav-BCL-2 transgenic mice, in which all hematopoietic cells overexpress BCL-2 and are resistant to apoptosis (Egle et al., 2004; Ogilvy et al., 1999), were inoculated with 9464D tumor and treated with low-dose CPM as above. In contrast to WT mice, intratumoral Treg cells from 9464D tumor bearing vav-BCL-2 mice were not depleted after low-dose CPM administration (Figures 4A and 4B). Furthermore, when comparing 9464D tumor growth after CPM administration, no significant difference in survival was observed in vav-BCL-2 mice, whereas a significant increase in survival was demonstrated in WT controls (Figure 4C). Overall, these data support the contention that depletion of Tregs is an important mechanism of action of low-dose CPM in this model. Although our data demonstrated that the only cell population assessed in wild type mice to reduce significantly in response to low dose CPM were intratumoral Treg, it should be noted as stated above, in this model Bcl2 is over expressed in all cells of hematopoietic origin and therefore other regulatory populations may being protected from death in this setting and their contributions cannot be fully excluded.

### Low-dose cyclophosphamide combined with anti-PD-1 antibody improves survival in neuroblastoma models

**Figure 5:**
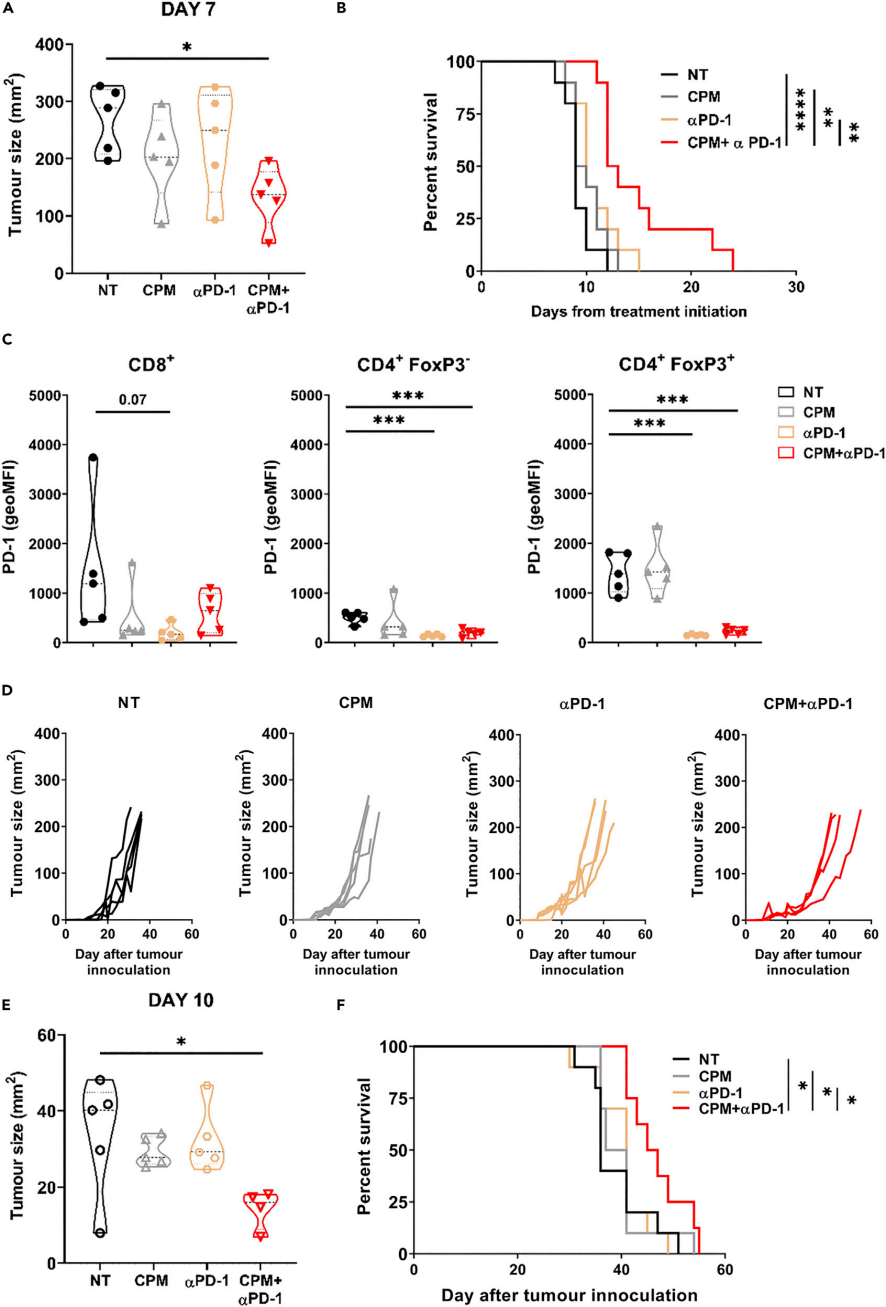
Combination of low-dose CPM with anti-PD-1 monoclonal antibody therapy increases survival in NXS2 and 9464D models *in vivo* (A) AJ mice bearing NXS2 subcut tumors were injected with either 40 mg/kg CPM or PBS i.p. (Day 0), followed by 250 μg of αPD-1 antibody or PBS at Day 3 + Day 6. Tumor growth was monitored. Example of tumor size comparison at Day 7 after CPM. (B) Survival analysis of A. (C) PD-1 expression on CD8^+^, FoxP3^-^ and FoxP3^+^ T cells demonstrated as geoMFI above background. For A–C example shown of two experiments with n = 5–10 per group. (D) 9464D mice were injected subcutaneously with 9464D cells. At palpable tumor size (Day 0) mice were treated as described in A. Tumor growth was recorded. (E) Example of tumor size comparison at Day 10 after CPM. (F) Survival analysis of D. For D–F example shown of two experiments with n = 5–10 per group (E–G). Data are represented as mean ±SD Significance was assessed by LogRank test (A) or t test (B–D and G) with ∗ = p <0.05, ∗∗ = p<0.01 and ∗∗∗ = p<0.01. See also Figures S8–S10.

After demonstrating low dose CPM’s immune modulatory properties in NB models, we investigated whether the drug could be combined therapeutically with immunomodulating mAb therapy. To that end, combination of CPM with either immune stimulatory (anti-4-1BB) or immune checkpoint blockade (anti-CTLA4 and anti-PD-1) mAb was assessed. For anti-PD-1, both wild type (WT) and Fc-null antibody (by deglycosylation or N297 mutation (Moreno-Vicente et al., 2022)) were used. This allows for assessment of the effects of PD-1 blockade both with and without effector function mediated by binding of FcγRs. It has previously been demonstrated that anti-PD-1 antibody therapy was improved when Fc function was abrogated (Dahan et al., 2015; Zhang et al., 2018). Although changes in Fc engagement can affect antibody half-life, deglycosylation did not impact the circulating concentration of antibody *in vivo* compared to the WT antibody (Figure S8). Using the NXS2 model, it was observed that combining CPM with either anti-CTLA-4 or anti-4-1BB mAb did not result in any significant changes in either tumor growth or survival over CPM alone (Figure S9). Although both anti-CTLA-4 and anti-4-1BB antibodies have been demonstrated to mediate therapy through intratumoral Treg depletion in pre-clinical models, this is used usually in the context of relatively small tumors (Buchan et al., 2018). In this model with large established tumors these antibodies are not therapeutic, and therefore are likely to not be effectively depleting Tregs. However, when combined with either WT (Figures S10A–S10C) or Fc-null (Figures 5A–5C) anti-PD-1 mAb tumor growth was slowed and a significant increase in survival was demonstrated compared to untreated mice. In addition, only CPM plus Fc-null anti-PD-1 resulted in an increase in survival compared to both antibody and CPM alone (Figure 5B). Furthermore, significant reduction in detectable PD-1 expression was observed on CD4^+^ T cell subsets, particularly Tregs (Figure 5C). This appeared to be as a result of anti-PD-1 treatment, as CPM treatment alone did not reduce expression of PD-1 on Tregs. When using the 9464D model, the same trend was seen, with CPM plus Fc-null anti-PD-1 treatment resulting in a significant slowing of tumor growth (Figures 5D, 5E, and S10D), and a significant increase in survival overall other treatment groups (Figure 5F). In this model however, no increase in survival over NT mice was seen with WT anti-PD-1 alone or in combination (Figures S10E and S10F). Similar results were obtained when mice were treated at a larger tumor size (5 × 5 mm), suggesting tumor burden may not impact combination therapy efficacy in this model (Figure S10G). Overall, these data demonstrate that low dose CPM can be combined with anti-PD-1 mAb to slow tumor growth and improve survival.

### Treg deletion by low-dose cyclophosphamide is transient

**Figure 6:**
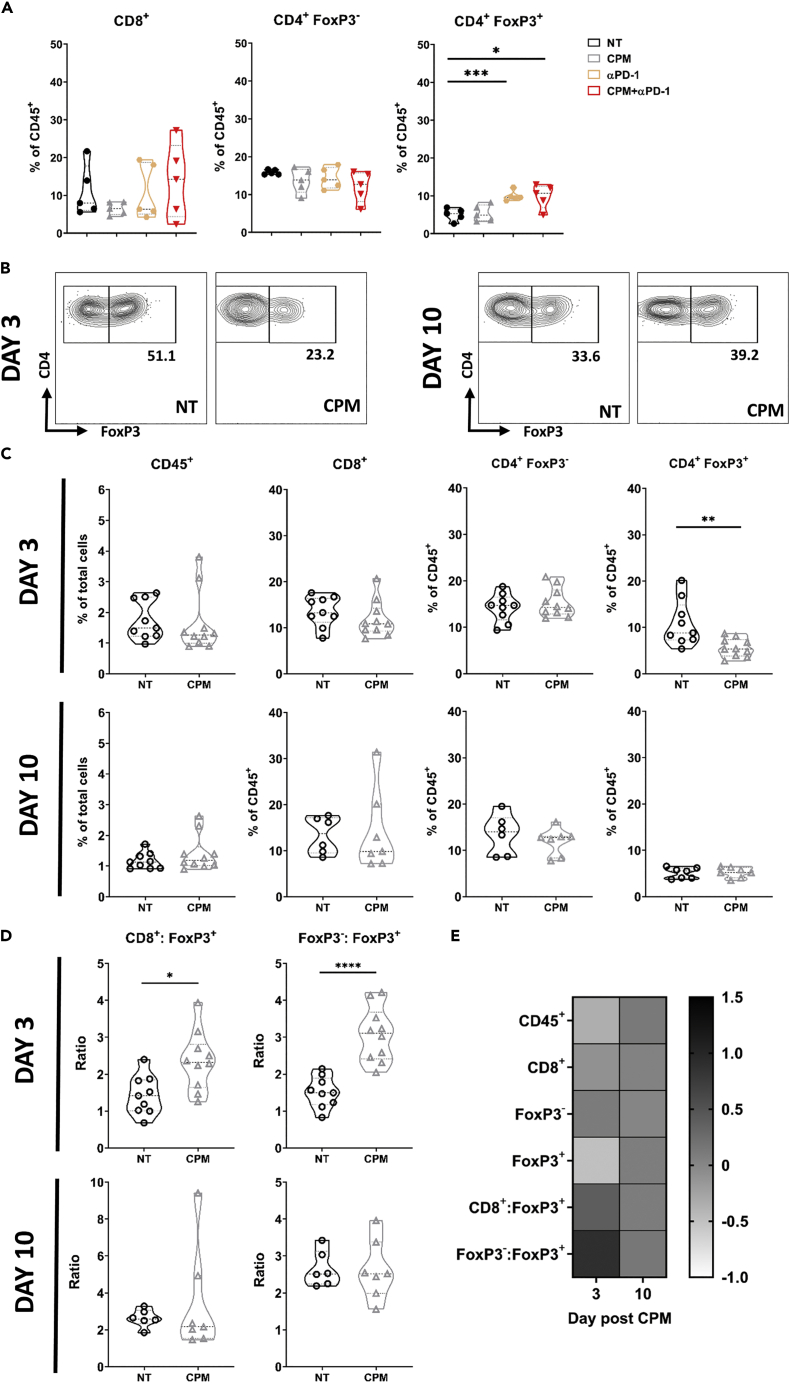
Depletion of Tregs by ‘low dose’ CPM is transient (A) AJ mice bearing NXS2 subcut tumors were injected with either 40 mg/kg CPM or PBS i.p (Day 0), followed by 250 μg of anti-PD-1 (Fc-null) antibody or PBS at Day 3 + Day 6. At Day 11 mice were culled and tumors were harvested for immunophenotyping by flow cytometry. Percentage of CD8^+^, CD4^+^ FoxP3+ and CD4^+^ FoxP3^+^ cells are demonstrated as a % of CD45^+^ cells. Data collected over two independent experiments, with n = 5 per group. (B) C57BL/6 mice bearing 9464D tumors were treated with 40 mg/kg CPM (Day 0). Tumors were harvested at either Day 3 or Day 10. Example contour plots of FoxP3 and CD4 expression, with percentages of FoxP3^+^ cells shown. (C and D) As in B with data of T cell populations in tumors shown as a percentage of total cells (CD45^+^) or percentage of CD45, and T cell ratios shown in D). Data collated from two independent experiments with n = 9 (NT) and n = 10 (CPM). (E) Summary heatmap of T cell populations in CPM treated tumors as fold change of NT. Data are represented as mean ±SD Significance was assessed by t-test with ∗ = p <0.05, ∗∗ = p <0.01, ∗∗∗ = p <0.001, ∗∗∗∗ = p<0.0001.

Although the above data demonstrated that CPM and anti-PD-1 therapy can significantly increase survival in these NB models, mice that received the dual treatment still eventually succumbed to tumor progression (Figure 5). As demonstrated above (Figure 2F), Tregs appeared to increase their expression of Ki67 after CPM, suggesting depletion is transient because of the increased proliferation of remaining Treg cells. When CPM was administered in conjunction with anti-PD-1 mAb in the NXS2 model, Treg percentages recovered and were significantly elevated compared to untreated mice at Day 11 after CPM (Figure 6A). A similar trend was seen in the 9464D model, with depletion of Tregs evident at Day 3 but not Day 10 after CPM (Figures 6B and 6C). Because of the Treg depletion, both CD8^+^:Treg and CD4^+^:Treg ratios were significantly increased over NT at Day 3, but not Day 10 (Figure 6D). Overall, as summarized in Figure 6E, these data demonstrate that the depletion of Tregs after a single low-dose CPM administration is transient, and Treg numbers are comparable to untreated tumors relatively shortly after dosing. Therefore, this suggested that to achieve sustained Treg depletion in these resistant neuroblastoma models, continued dosing of CPM was likely required.

### Metronomic low-dose cyclophosphamide in combination with anti-PD-1 mAb improves survival

**Figure 7:**
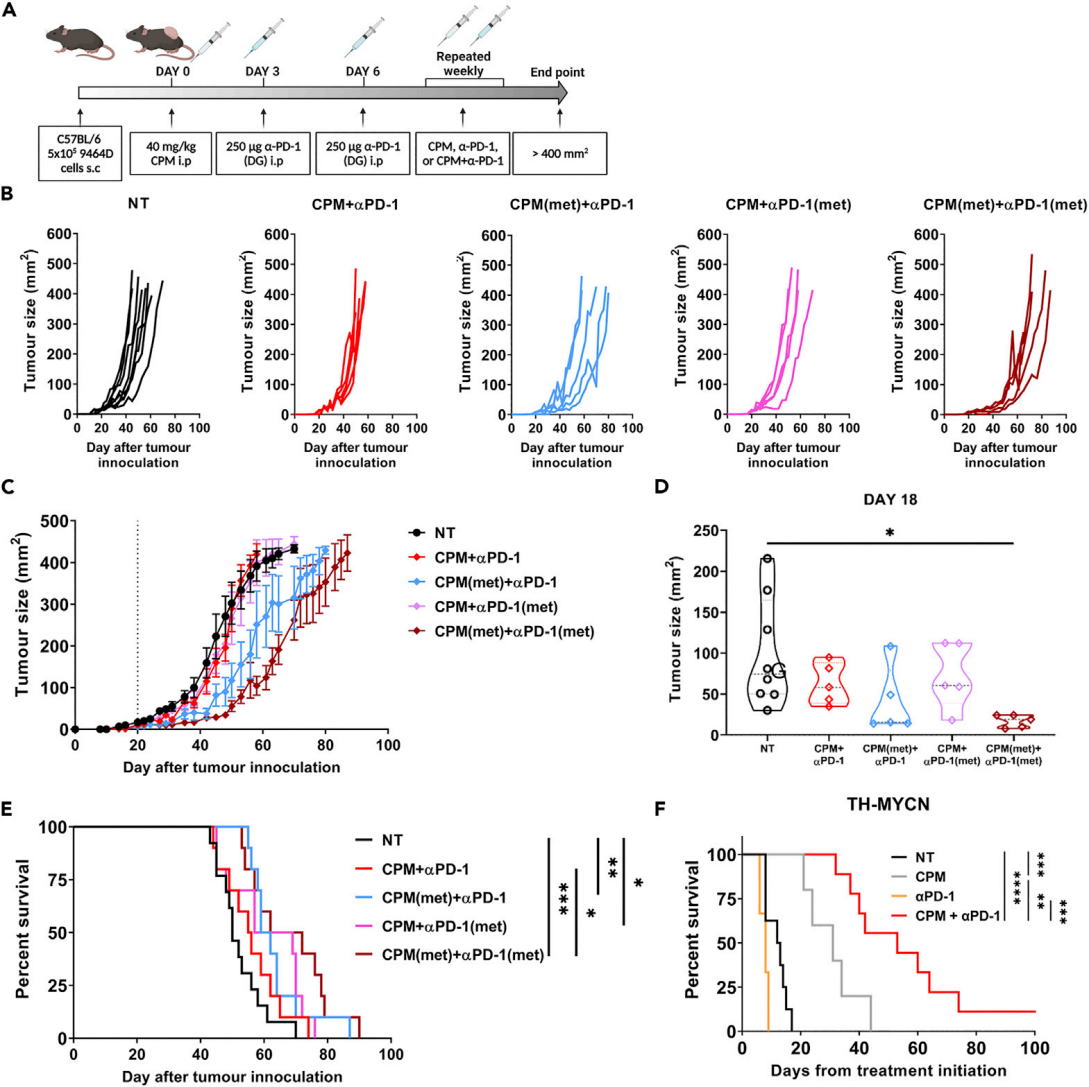
Improved efficacy with CPM and anti-PD-1 combination therapy in 9464D and TH-MYCN models (A) C57BL/6 mice bearing 9464D subcut tumors were treated with 40 mg/kg CPM i.p or PBS (Day 0). Day 3 + 6 mice were treated with 250 μg of anti-PD-1 (Fc-null) antibody i.p. Mice were then treated on a weekly basis with either 40 mg/kg CPM, 150 μg anti-PD-1 or both CPM and anti-PD-1, until endpoint was reached. Experiment schematic is demonstrated, created in Biorender. (B and C) Tumor growth curves of each group (B), with average tumor growth shown in C. Dotted line denotes treatment start point at Day 20 where tumors were ∼5 × 5 mm. (D) Example tumor size comparison between groups at Day 18. (E) Survival curve of all groups. Example shown of two independent experiments (B-D) with n= 8 (NT), n = 5 (All other groups). Combined data from two experiments shown in E with n = 10–15 per group. (F) Heterozygous TH-MYCN mice with ∼1 cm palpable tumors were injected with either 40 mg/kg CPM or PBS i.p (Day 0). On Day 3 + 6 either 250 μg of anti-PD-1 (Fc-null) antibody or PBS was given i.p. Mice were monitored until endpoint where survival curve was generated. Data points represent one experiment collected over time because of spontaneity of tumor development, confirmatory of the data collected with the 9464D (E). n= 3–9 per group. Data are represented as mean ±SD Significance was assessed by t test (D) or LogRank test (E + F) with ∗ = p <0.05, ∗∗ = p <0.01, ∗∗∗ = p <0.001, ∗∗∗∗ = p<0.0001.

As CPM dependent depletion of Tregs was demonstrated to be short-lived (Figure 6), metronomic low dose CPM was investigated (Wu and Waxman, 2018), with 40 mg/kg of CPM administered weekly to 9464D tumor bearing mice (Figure 7A). This schedule, in combination with anti-PD-1, was more effective at slowing tumor growth (Figures 7B–7D) and significantly improved survival compared to single dose CPM + PD-1 therapy (Figure 7E). These data demonstrate that metronomic dosing of CPM together with immunotherapy could be utilized to improve survival in more resistant settings.

### Combination of low dose CPM with anti-PD-1 leads to increased survival in a spontaneous murine neuroblastoma model

Previous work has demonstrated that the transgenic TH-MYCN model of neuroblastoma is receptive to combination therapy, and has a more comparable immune infiltrate to human disease, relative to subcutaneous models (Webb et al., 2020). As these mice spontaneously develop neuroblastoma, treatment was started once tumors were approximately 1 cm in diameter. Single dosing of CPM resulted in a significant increase in survival over NT, suggesting TH-MYCN tumors were more sensitive to CPM monotherapy than subcutaneous models (Figure 7F). However, survival was further increased with the combination of low dose CPM with anti-PD-1 mAb, with some mice showing no signs of recurrence at Day 100 after treatment initiation. These data support the therapeutic effect of combination low dose CPM and anti-PD-1 observed in the subcutaneous models, and provides evidence that this combination strategy could potentially be translated into clinical trials.

## Discussion

Outcome for high risk neuroblastoma remains poor despite intensive and highly toxic treatment regimens. Novel approaches, which can be used in conjunction with, or even allow reduction of, current therapies, are needed improve survival and reduce toxicity. Although chemotherapy agents are given primarily for their direct cytotoxic effects, a number of drugs, including CPM, have been demonstrated to have wide ranging immunomodulatory effects (Abu Eid et al., 2016, Hughes et al., 2018, Scurr et al., 2017, Wu and Waxman, 2018). Immunomodulatory mAb therapies have fast become part of standard treatment protocols in many adult cancer types. mAbs targeting checkpoint inhibitor molecules on the surface of activated T cells, have demonstrated striking objective responses in some patients with melanoma and non-small cell lung cancer (Rizvi et al., 2015; Robert et al., 2019). However, these therapies have limited efficacy as single agents, particularly in pediatric cancers (Davis et al., 2020; Geoerger et al., 2020). Combination with other immunotherapies or standard treatment is therefore needed to unlock their full potential. To that end, we investigated underlying mechanisms of CPM and anti-PD-1 combination therapy in the context of murine neuroblastoma models.

CPM, among other chemotherapies, has been reported to induce ICD mechanisms in a number of cancer settings (Vanmeerbeek et al., 2020; Du and Waxman, 2020; Gebremeskel et al., 2017), but there is minimal data regarding ICD in the context of neuroblastoma (Inoue et al., 2014, 2017). Therefore, we chose to investigate whether CPM can induce ICD in neuroblastoma models. We demonstrated that although there was some modulation of certain ICD markers, the changes were relatively subtle (Figure 1). However, observable changes in HMGB1 expression and location within the cell was apparent. Although release of ICD modulators such as HMGB1 could lead to the influx of myeloid cells because of their chemoattractive properties (Ahmed and Tait, 2020), the modest effects observed here suggest that this is unlikely to be a major factor contributing to the therapeutic efficacy of low-dose CPM in neuroblastoma models. Despite an observable increase in activation associated FcγRs, such as FcγRI (Junker et al., 2020), (Figure S6), few changes in numbers of myeloid cells after CPM (Figure S4) were demonstrated. This indicated that these cells were not induced to migrate into the TME by CPM treatment.

To better understand whether immune mediated mechanisms were responsible for the efficacy of low-dose CPM, immunophenotyping of CPM treated tumors was conducted (Figure 2). Here, it was observed that low-dose CPM led to a significant decrease in Tregs, specifically within the TME, while not affecting effector cell numbers. The ability of CPM to deplete Tregs in tumors has been demonstrated previously in other tumor types (Dimeloe et al., 2014; Hughes et al., 2018; Huijts et al., 2019). Although, one study has also demonstrated CPM ability to deplete Tregs in a GD2-negative murine neuroblastoma model (AgN2a), this was only investigated in the spleen and not the tumor (Gershan et al., 2015). As Treg cells are capable of suppressing anti-tumor T cell responses, a decrease in their number could potentially enhance either endogenous anti-neuroblastoma immunity or response to immunotherapy. Of interest, the depletion of Treg cells was only observed within the TME. This was due to increased levels of proliferation in tumor infiltrating Tregs, as demonstrated by increased Ki67 expression compared to other tissues (Figure 2F). Alkylating agents, such as CPM, are known to be cytotoxic to highly proliferating cells, such as cancer cells. Therefore, if tumor Treg cells have higher levels of Ki67 which is indicative of proliferation, then this could suggest why these cells appeared to be selectively depleted by CPM from the TME. Furthermore, Tregs have been previously demonstrated to have sensitivity to CPM because of low expression patterns of drug efflux transporters such as ABCB1 (Dimeloe et al., 2014). Recent studies have also suggested a role for interferon regulatory factor-1 in CPM induced Treg depletion (Buccione et al., 2018). Although no significant changes in effector cell (CD8^+^ and CD4^+^ FoxP3^-^) populations were observed, further analysis of these subsets demonstrated changes in phenotype (Figure 3). Increased expression of activation markers such as CD107a, at day 3 was seen in both CD8 and CD4 effectors, with an increase in exhaustion related markers, such as TIM-3, at day 10 after CPM. This could suggest that the depletion of Tregs by CPM has lifted the suppression of effector cells at the early time points after CPM.

As the majority of immune modulation capacity appeared to be driven by Treg depletion, further analysis of the Treg phenotype after CPM was conducted (Figure 3). Here, a decrease in markers such as CD25 and OX40 after CPM, suggest that the Tregs remaining within the tumor could have reduced suppressor function compared to NT tumor Tregs (Fontenot et al., 2003; Willoughby et al., 2017). Interestingly, an increase in Ki67 was also seen in Tregs after CPM compared to controls, suggesting greater proliferation of the remaining cells. The importance of Treg depletion as a mechanism of action for low dose CPM was investigated by using apoptosis resistant mice (Figure 4). Here, tumor Tregs in vav-BCL-2 mice were not depleted after CPM administration compared to WT mice, leading to a decrease in survival. This suggests that CPM is able to deplete Tregs via classical apoptosis, and that it is the reduction in Treg numbers which is driving the therapeutic benefit of CPM in these neuroblastoma models.

Owing to the immunomodulatory capacity of CPM demonstrated previously, combination with mAb therapy was assessed *in vivo*. It was demonstrated that the combination of CPM with anti-PD-1 mAb resulted in an increase in survival (Figures 5, S9, and S10) in both neuroblastoma models. Rossi et al., has previously demonstrated combination CPM with anti-PD-L1/2 antibodies using the EG7-OVA model, however a much larger dose (100 mg/kg) was used (Rossi et al., 2020). Furthermore, although studies have also demonstrated efficacy of low-dose CPM combined with anti-PD-1 (Weir et al., 2016) or anti-PD-L1 antibody treatment (Orecchioni et al., 2018) neither of these studies were conducted in the context of NB. In addition, CPM was given continuously, and was cumulatively higher than the dosing given here. Lastly, Mkrtichyan et al. also reported the same combination efficacy but in an HPV antigen expressing tumor model (Mkrtichyan et al., 2011). The combination therapy data presented here builds on these previous studies and demonstrates that this treatment strategy could be applied in neuroblastoma, and possibly other solid tumor settings. We noted that an Fc-null version of the PD-1 mAb resulted in increased efficacy over its WT counterpart, in keeping with previous reports in other models (Dahan et al., 2015; Moreno-Vicente et al., 2022). As we demonstrated changes in FcγR on myeloid populations within CPM treated tumors (Figure S6), an Fc-null version of the mAb may prevent it from binding these FcγRs and deleting populations of PD-1^+^ effector cells via antibody dependent cellular phagocytosis, impairing an anti-tumor immune response (Liu et al., 2020; Dahan et al., 2015; Moreno-Vicente et al., 2022). Therefore, as an Fc-null antibody has no effector function, there would be negligible depletion of Tregs by anti-PD-1 antibody dependent methods. Furthermore, we observed a reduction in PD-1 on T cell subsets after anti-PD-1 treatment. This was mainly apparent on CD4^+^, and particularly Treg cells (Figure 5C). Although this downregulation could demonstrate exhaustion of effector cells, because of PD-1 upregulation after activation (Jubel et al., 2020), this is unlikely as the tumors responded to anti-PD-1 treatment. One explanation could be receptor mediated endocytosis, leading to a downregulation of cell surface expression of PD-1 after antibody engagement (Ritchie et al., 2013). It is important to note that despite the increase in survival, CPM + anti-PD-1 therapy did not lead to tumor clearance in either model. This may be because of the rebound of Treg numbers (Figure 6) seen after CPM at later time points (>10 days), demonstrating that Treg depletion by CPM is transient. This is supported by evidence of increased proliferation of Treg cells after CPM (Figure 3). Therefore, metronomic dosing was incorporated into the combination therapy of CPM with anti-PD-1 (Figure 7). Here, it was demonstrated that weekly dosing of both CPM and anti-PD-1 significantly increased survival over the single CPM + anti-PD-1 arms in 9464D model. This suggests that metronomic administration of low dose CPM is required to maintain depletion of Tregs within the tumor environment. Repetitive administration of low dose chemotherapy, so called ‘metronomic’ dosing, has been shown previously to improve the immunogenic effect in numerous cancer models (Wu and Waxman, 2018; Peng et al., 2013; Orecchioni et al., 2018; Weir et al., 2016). Furthermore, recent trials have also shown promise when metronomic CPM and immunotherapy are combined in adult cancer settings (Zsiros et al., 2021; Toulmonde et al., 2018; Le Cesne et al., 2019). Finally, we demonstrated that the combination of CPM with anti-PD-1 antibody therapy significantly increased survival in the TH-MYCN spontaneous model of neuroblastoma (Figure 7). Although this model appears to be inherently more sensitive to CPM alone compared to the cell line models, the combination therapy was able to lead to greatly extended survival over CPM alone. Single agent anti-PD-1 therapy has not shown clinical efficacy in the majority of pediatric cancers, likely because of the immunologically ‘cold’ microenvironment of these tumors. It is therefore likely that combinational approaches will be needed to achieve therapeutic success using immune checkpoint blockade in neuroblastoma. To date, there are limited clinical trials investigating the combination of PD-1 and cyclophosphamide in neuroblastoma. The ESMART trial (Pasqualini et al., 2021) included an arm investigating metronomic CPM combined with nivolumab. Although this study did not demonstrate significant activity, only 2 of the 13 patients had neuroblastoma, and the recruited patients are likely to have been heavily pre-treated. A further ongoing trial (NCT03585465) is also investigating this combination, however this includes additional chemotherapy agents (vinblastine and capecitabin), and again the study is recruiting all pediatric tumors. Because the immunological effects observed in this study may not be generalizable to other pediatric cancers, and a study specifically in patients with neuroblastoma may be required to show benefit. In addition, traditional early phase trials conducted in patients who have exhausted all other treatment options may not be optimal for testing this type of chemo-immunotherapy, and alternative trial designs may need to be considered.

Overall, we demonstrated the immunomodulatory capacity of ‘low-dose’ CPM in murine models of neuroblastoma, and its efficacy in combination with anti-PD-1 mAb. As CPM is widely used in current neuroblastoma treatment regimens, and anti-PD-1 therapy is being investigated in clinical trials, we hope that these data provide evidence to support testing this combination therapy in a clinical setting.

### Limitations of the study

Although three distinct murine neuroblastoma models have been used here, the majority of the study was performed using subcutaneous models, which may not fully recapitulate the tumor microenvironment found in human neuroblastoma. To reduce this limitation, two different subcutaneous models were used alongside a spontaneous transgenic model.

## STAR★Methods

### Key resources table

**Table:** 

REAGENT or RESOURCE	SOURCE	IDENTIFIER
**Antibodies**		

Anti-mouse 4-1BB (LOB 12.0) Mouse IgG1	In house; Buchan et al., Immunity 2018	N/A
Anti-mouse 4-1BB (LOB 12.0) Mouse IgG2a	In house; Buchan et al., Immunity 2018	N/A
Anti-mouse CTLA-4 (UC10 F41011) Mouse IgG1	In house	N/A
FITC Anti-mouse Fc Block (2.4G2) Fab_2_	In house; Daeron et al., European Journal of Immunology 1986	N/A
FITC Anti-mouse FcγRI (AT152) Fab_2_	In house; Tutt et al., Journal of Immunology 2015	N/A
FITC Anti-mouse FcγRIIb (AT130-2) Mouse IgG1	In house; Tutt et al., Journal of Immunology 2015	N/A
FITC Anti-mouse FcγRIII (AT154) Fab_2_	In house; Tutt et al., Journal of Immunology 2015	N/A
FITC Anti-mouse FcγRIV (AT137) Fab_2_	In house; Tutt et al., Journal of Immunology 2015	N/A
Anti-mouse PD-1 (EW1.9) Rat IgG	In house; Buchan et al., Immunity 2018	N/A
Anti-mouse PD-1 (EW1-9) Fc-null (deglycosylated or N297A mutant)	In house; oreno et al., Journal of ImmunoTherapy of Cancer 2022	N/A
APC Anti-mouse 4-1BB (17B5) Syrian Hamster IgG	Thermo Fischer	Cat#17-1371-82; RRID:AB_2573162
PerCP-Cy5.5 Anti-mouse/human B220 (RA3-6B2) rat IgG2a, κ	Biolegend	Cat#103235; RRID:AB_893356
AlexaFluor 488 Anti-mouse CD107a (eBio1D4B) rat IgG2a, κ	Thermo Fischer	Cat#53-1071-82; RRID:AB_657536
PE Anti-mouse CD11b (M1/70) rat IgG2b, κ	Thermo Fischer	Cat#12-0112-82; RRID:AB_2734869
eFluor 450 Anti-mouse CD11c (N418) Armenian Hamster IgG	Thermo Fischer	Cat#48-0114-82; RRID:AB_1548654
APC Anti-mouse CD19 (1D3) rat IgG2a, κ	Thermo Fischer	Cat#17-0193-82; RRID:AB_1659676
APC CD25 (PC61) rat IgG1, λ	Biolegend	Cat#102011; RRID:AB_312860
Pacifc Blue Anti-mouse CD3e (500A2) Syrian Hamster IgG2	BD Biosciences	Cat# 558214; RRID:AB_397063
eFluor 450 Anti-mouse CD4 (GK1.5) rat IgG2b, k	Thermo Fischer	Cat#48-0041-82; RRID:AB_10718983
FITC Anti-mouse CD4 (RM4-5) rat IgG2a	Thermo Fischer	Cat#11-0042-82; RRID:AB_464896
FITC Anti-mouse CD44 (IM7) rat IgG2b	Thermo Fischer	Cat#11-0441-82; RRID:AB_465045
PerCP-Cy5.5 Anti-mouse CD44 (IM7) rat IgG2b	Biolegend	Cat#103031; RRID:AB_2076206
PE Anti-mouse CD44 (IM7) rat IgG2b	Thermo Fischer	Cat#12-0441-82; RRID:AB_465664
V450 Anti-mouse CD45.2 (104) mouse IgG2a	BD Biosciences	Cat#560697; RRID:AB_1727495
PE-Cy7 Anti-mouse CD45.2 (104) mouse IgG2a	BD Biosciences	Cat#560696; RRID:AB_1727494
PE Anti-mouse CD49b (DX5) rat IgM	BD Biosciences	Cat#553858; RRID:AB_395094
APC-Cy7 Anti-mouse CD62L (MEL-14) rat IgG2a, κ	Biolegend	Cat#104427; RRID:AB_830798
PE-Cy7 Anti-mouse CD62L (MEL-14) rat IgG2a, κ	BD Biosciences	Cat#560516; RRID:AB_1645257
PerCP-Cy5.5 Anti-mouse CD8a (53-6.7) rat IgG2a	Thermo Fischer	Cat#45-0081-82; RRID:AB_1107004
APC Anti-mouse CD8a (53-6.7) rat IgG2a	Thermo Fischer	Cat#17-0081-82; RRID:AB_469335
PE Anti-mouse Calreticulin (FMC75) mouse IgG1	Abcam	Cat#ab83220; RRID:AB_1859755
PerCP-Cy5.5 Anti-mouse CTLA4 (UC10-4B9) Armenian Hamster IgG	Biolegend	Cat#106315; RRID:AB_2564473
AlexaFluor 488 Anti-mouse EOMES (Dan11mag) rat IgG2a, κ	Thermo Fischer	Cat#53-4875-82; RRID:AB_10854265
PE-Cy7 Anti-mouse EOMES (Dan11mag) rat IgG2a, κ	Thermo Fischer	Cat#25-4875-82; RRID:AB_2573454
APC Anti-mouse F4/80 (Cl:A3-1) rat IgG2b	BioRad	Cat#MCA497; RRID:AB_2098196
PE Anti-mouse Foxp3 (FJK16s) rat IgG2a	Thermo Fischer	Cat#12-5773-82; RRID:AB_465936
FITC Anti-human/mouse Granzyme B (GB11) mouse IgG1, κ	Biolegend	Cat#515403; RRID:AB_2114575
AlexaFluor 647 Anti-mouse Hsp-70 (ERP16892) rabbit IgG	Abcam	Cat#ab204691; RRID:AB_2910093
V500 Anti-mouse I-A/I-E (M5/114) rat IgG2b, κ	BD Biosciences	Cat#562366; RRID:AB_11153488
FITC Anti-mouse IFNγ (XMG1.2) rat IgG1, κ	Thermo Fischer	Cat#11-7311-41; RRID:AB_10718840
FITC Anti-mouse Ki67 (16A8) rat IgG2a, κ	Biolegend	Cat#652409; RRID:AB_2562140
PerCP-Cy5.5 Anti-mouse Ki67 (16A8) rat IgG2a, κ	Biolegend	Cat#652423; RRID:AB_2629530
PE-Cy7 Anti-mouse Ki67 (16A8) rat IgG2a, κ	Biolegend	Cat#652425; RRID:AB_2632693
APC Anti-mouse KLRG1 (2F1) Syrian Hamster IgG	Thermo Fischer	Cat#17-5893-82; RRID:AB_469469
APC Anti-mouse LAG3 (eBioC9B7W) rat IgG1, κ	Thermo Fischer	Cat#17-2231-82; RRID:AB_2573184
PerCP-Cy5.5 Anti-mouse Ly6C (HK1.4) rat IgG2c, κ	Thermo Fischer	Cat#45-5932-82; RRID:AB_2723343
PE-Cy7 Anti-mouse Ly6G/Ly6C (RB6-8C5) rat IgG2b, κ	Thermo Fischer	Cat#25-5931-82; RRID:AB_469663
PE Anti-mouse NKG2ACE (20d5) rat IgG2a	Novus	Cat#NBP1-28100PE; RRID:AB_1853413
APC Anti-mouse OX40 (OX-86) rat IgG1, κ	Thermo Fischer	Cat#17-1341-82; RRID:AB_10717260
APC Anti-mouse PD-1 (RMP1-30) rat IgG2a, κ	Thermo Fischer	Cat#17-9981-82; RRID:AB_10852564
APC Anti-mouse PD-1 (29F.1A12) rat IgG2a, κ	Biolegend	Cat#135209; RRID:AB_2251944
PE- Anti-mouse PD-L1 (10F.9G2) rat IgG2a, λ	Biolegend	Cat#124307; RRID:AB_2073557
APC Anti-mouse Tbet (4B10) mouse IgG1, κ	Biolegend	Cat#644813; RRID:AB_10896913
PerCP-Cy5.5 Anti-mouse Tbet (4B10) mouse IgG1, κ	Biolegend	Cat#644805; RRID:AB_1595593
FITC Anti-mouse TIM3 (RMT3-23) rat IgG2a, κ	Thermo Fischer	Cat#11-5870-82; RRID:AB_2688129
Anti-mouse HMGB1 (EPR3507) rabbit IgG	Abcam	Cat#ab216986; RRID:AB_1603373

**Chemicals, peptides, and recombinant proteins**		

Mafosfamide	Santa Cruz	Cat#sc-211761; CAS84211-05-2
Cyclophosphamide	Sigma-Aldrich	Cat#PHR1404; CAS6055-19-2
Doxorubicin	Sigma-Aldrich	Cat#PHR1789; CAS25316-40-9

**Critical commercial assays**		

eBioscience™ Foxp3 / Transcription Factor Staining Buffer Set	Thermo Fischer	Cat#00-5523-00
ImmPRESS™ HRP Anti-Rabbit IgG Polymer Detection Kit	Vector Labs	Cat#MP-7401-15
Vector® NovaRED® Substrate Kit, Peroxidase (HRP)	Vector Labs	Cat#SK-4800
Cell Titer 96 non-radioactive cell proliferation assay	Promega	Cat#G4000
Live/Dead Fixable Aqua Dead Cell Stain kit	Thermo Fischer	Cat#L34957

**Experimental models: cell lines**		

NXS2 (murine neuroblastoma)	Prof. Dr. Holger Lode, Medical University of Greifswald	N/A
9464D (murine neuroblastoma)	Dr Rimas Orentas, NIH	N/A

**Experimental models: Organisms/strains**		

TH-MYCN	Originally provided by William Weiss, NIH. Bred and maintained at University of Southampton	Developed by Wiess et al., The EMBO journal 1997
Vav-Bcl2	Originally provided by, Andreas Strasser (Melbourne, Australia). Bred and maintained at University of Southampton	Developed by Ogilvy et al., PNAS 1999
C57BL/6J	Bred and maintained at University of Southampton	RRID:IMSR_JAX:000664
A/J	Bred and maintained at University of Southampton	RRID:IMSR_JAX:000646

**Software and algorithms**		

FlowJo version 10.6.2	Tree Star	https://www.flowjo.com/; RRID:SCR_008520
HALO image analysis software (v3.1.1076.451), Multiplex IHC v2.3.4 module	Indica	https://www.indicalab.com/halo/; RRID:SCR_018350
GraphPad Prism 9	GraphPad Software, La Jolla, California USA	https://www.graphpad.com:443/; RRID: SCR_002798
FACS DIVA version 8	BD Biosciences	https://www.bdbiosciences.com/en-gb/products/software/instrument-software/bd-facsdiva-software

**Other**		

PNGase F	Promega	Cat#V4831
Liberase TL	Merck (Roche)	Cat#V4831
DNAse	Sigma-Aldrich	Cat#10104159001
100 μm cell strainer	Corning	Cat#352360
Red cell lysis buffer	Bio-Rad	Cat#BUF04C
VectaMount® Permanent Mounting Medium	Vector Labs	Cat#H-5000-60
VECTASHIELD® HardSet™ Antifade Mounting Medium	Vector Labs	Cat#H-1400-10

### Resource availability

#### Lead contact

Further information and requests for resources and reagents should be directed to and will be fulfilled by the lead contact, Stephen Beers (s.a.beers@soton.ac.uk).

#### Materials availability

This study did not generate new unique reagents.

### Experimental model and subject details

#### Mice

Mice were bred and maintained in local facilities and experiments approved by the local ethical committee under Home Office licenses RRF30/2964, PB24EEE31 and P81E129B7. Experiments conformed to the Animal Scientific Procedure Act (UK). All animal experiments had ethical approval from the University of Southampton Animal Welfare and Ethics Review Board (AWERB). A/J and C57BL/6 female mice were bred locally at the University of Southampton. TH-MYCN transgenic mice were developed by Weiss et al. (1997). Heterozygous (het) mice are used to allow for full development of the immune system before tumor appearance. Female (<12 weeks old) Vav-B cell lymphoma 2 (BCL-2) transgenic mice on C57BL/6 background were developed by Ogilvy et al., and have hematopoietic cells resistance to apoptosis (Egle et al., 2004; Ogilvy et al., 1999). All animals were included in analysis unless otherwise stated.

#### Antibodies

In-house: In-house produced monoclonal antibodies (mAbs) for *in vivo* and flow cytometry experiments are listed in key resources table. Quality control of mAbs was conducted as detailed previously (Buchan et al., 2018). Deglycosylation was conducted using PNGase F enzyme (Promega) and confirmed by electrophoresis. Commercially sourced antibodies: Antibodies used throughout flow cytometry, immunohistochemistry and immunofluorescence experiments were purchased from various commercial sources as detailed in key resources table.

#### Murine neuroblastoma cell-lines and chemotherapies

GD2-expressing NXS2 cell line (provided by Prof. Dr. Holger Lode, Medical University of Greifswald, Germany), and 9464D cells (kindly provided by Dr Rimas Orentas, NIH) were cultured as detailed previously (Webb et al., 2020). 2 x 10^6^ NXS2 cells were injected subcutaneously into recipient A/J mice, and 5 × 10^5^ cells were injected subcutaneously into recipient C57BL/6 mice. Mice were randomly assigned to treatment groups, to maintain similar average tumor size between groups. For *in vivo* use: Cyclophosphamide and Doxorubicin (DOX) were purchased from Sigma. Drug was administered to the mice via intraperitoneal injection. For *in vitro* use: Metabolically active CPM derivative mafosfamide (MAF; Santa Cruz), was used *in vitro* (Schwartz and Waxman, 2001). Chemotherapy agent was used at concentrations described in the figure legends. Treatments: Mice were treated with either CPM or PBS on day 0. At day 3 and 6, mice were administered with either anti-PD-1 (EW1-9), anti-CTLA4 (UC10 F41011) or anti-4-1BB (Lob 12.0) at 250 μg in sterile PBS as detailed in experiments.

### Method details

#### Cell death assay

Cell Titer 96 non-radioactive cell proliferation assay (Promega) was carried out according to manufacturer’s instructions in 96-well plates. Absorbance was read at 530 nm with Epoch microplate reader (Biotek) after 1 h.

#### Tissue dissociation

At time points detailed in experiments, mice were culled, and tumors, spleens and lymph nodes harvested. Spleens and lymph nodes were mechanically disassociated. Tumors were incubated with Liberase TL (Roche) with DNAase. All tissues were passed through 100 μm cell strainer. Cells were re-suspended in 1% PBS/BSA and counted using Coulter Counter (Beckman).

#### Flow cytometry

Live/dead staining: Live/Dead Fixable Aqua Dead Cell Stain kit (Invitrogen) was used as per manufacturer’s instructions, as detailed. Extracellular staining: Fc-Blocking antibody (2.4G2) was added to 1 × 10^6^ cells, followed by staining with antibody master mixes. Cells were fixed and red cells lysed using red cell lysis buffer (Bio-Rad). Intracellular staining: FoxP3 Staining Buffer set (eBioscience) was used, and manufacturer’s instruction followed, fixation/permeabilization carried out overnight, before addition of intracellular antibody mixes. Analysis and data collection: Data was collected on FACS Canto II or FACS Caliber (BD Biosciences) as stated. Analysis was conducted using FACS DIVA version 8 (BD Biosciences) or FlowJo version 10.6.2 (Tree Star).

#### Ecto-CRT and HSP-70 analysis

Cells were plated into a 96-well plate and left to adhere. Cell death was induced with addition of chemotherapy agent at stated dose for 24 h, after which cells were harvested and stained for immunogenic cell death markers using flow cytometry.

#### PBMC collection

Blood was collected via tail vein at time points detailed in individual experiments. Antibodies used for flow cytometry staining were added directly.

#### HMGB1 immunohistochemistry

Day 3 after CPM administration 9464D tumors were harvested, fixed in 4% formalin, and embedded in paraffin with 8 μm sections cut using microtome. Sections were deparaffinized then rehydrated. EDTA (pH 9) antigen retrieval was carried out and blocked with 2.5% horse serum. HMGB1 antibody (as detailed in Table S2) was added and ImmPRESS HRP Anti-Rabbit IgG Polymer Detection Kit (Vector Labs) was used. Sections were then incubated in Vector NovaRed-horse radish peroxidase (Vector Labs), counterstained with hematoxylin, and dehydrated by increasing alcohol concentrations. Sections were washed in Histo-Clear, and mounted using VectaMount (Vector Labs). Quantification: Slides were digitized using Olympus dotSlide. They were interpreted by a consultant pathologist and analyzed using Indica’s HALO image analysis software (v3.1.1076.451), Multiplex IHC v2.3.4 module. Strong, moderate and weak intensity was set arbitrarily by the interpreting pathologist, and settings were kept consistent for all cases analyzed.

#### HMGB1 immunofluorescence

Coverslips were coated with poly-L-lysine, and 20,000 9464D cells were seeded overnight, with MAF (75 ug/ml) added for 24 h. Coverslips were washed and fixed in 4% PFA, followed by permeabilization with 0.25% Triton X-100 and blocking in 2% BSA in PBS +0.05% Tween. HMGB1 antibody was added at 1:350, and anti-rabbit Alexa Fluor 488 secondary antibody was used at 1:1000. Coverslips were mounted using Vectashield Hardset (Vector Labs). Imaging: Laser scanning confocal microscopy was conducted using Leica TCS SP5 inverted microscope with environmental chamber (Leica), using oil ×40 objectives.

#### ELISA of antibody serum concentrations

Mice were injected with either 100 μg or 500 μg dose of antibody by i.p. and bled at time points detailed in figure legends. Serum was collected by centrifugation. ELISA plates were coated with goat anti-rat antibody (in house), then blocked with 1% BSA-PBS. Standards and samples were diluted 2-fold across the plate. After washing, sheep anti-rat HRP antibody (in house) was added. Plate was washed, substrate mix added and incubated, reaction was stopped with 2.5 M H_2_SO_4_, and then the plate read on Epoch microplate reader at 450 nm.

### Quantification and statistical analysis

#### Statistical analysis

Graphs were produced and statistical analyses performed using GraphPad Prism version 9 (GraphPad) and Excel 2013 (Microsoft Corporation). Statistical details and analysis used can be found in the figure legends. Differences were considered significant when p-value < 0.05.

## Data Availability

• All data reported in this paper will be shared by the lead contacton request. • This paper does not report original code. • Any additional information required to reanalyze the data reported in this paper is available from the lead contacton request.
