# Supplementary material for: Cyclophosphamide depletes tumor infiltrating T regulatory cells and combined with anti-PD-1 therapy improves survival in murine neuroblastoma
Source: iScience. 2022 Aug 23;25(9):104995. doi: 10.1016/j.isci.2022.104995 (PMC9463572; doi:10.1016/j.isci.2022.104995)
Supplement: Document S1. Figures S1–S — 10 [file mmc1.pdf]

iScience, Volume *25*

Supplemental information

Cyclophosphamide depletes tumor infiltrating T regulatory cells and combined with anti-PD-1 therapy improves survival in murine neuroblastoma

Emily R. Webb, Julia Moreno-Vincente, Alistair Easton, Silvia Lanati, Martin Taylor, Sonya James, Emily L. Williams, Vikki English, Chris Penfold, Stephen A. Beers, and Juliet C. Gray

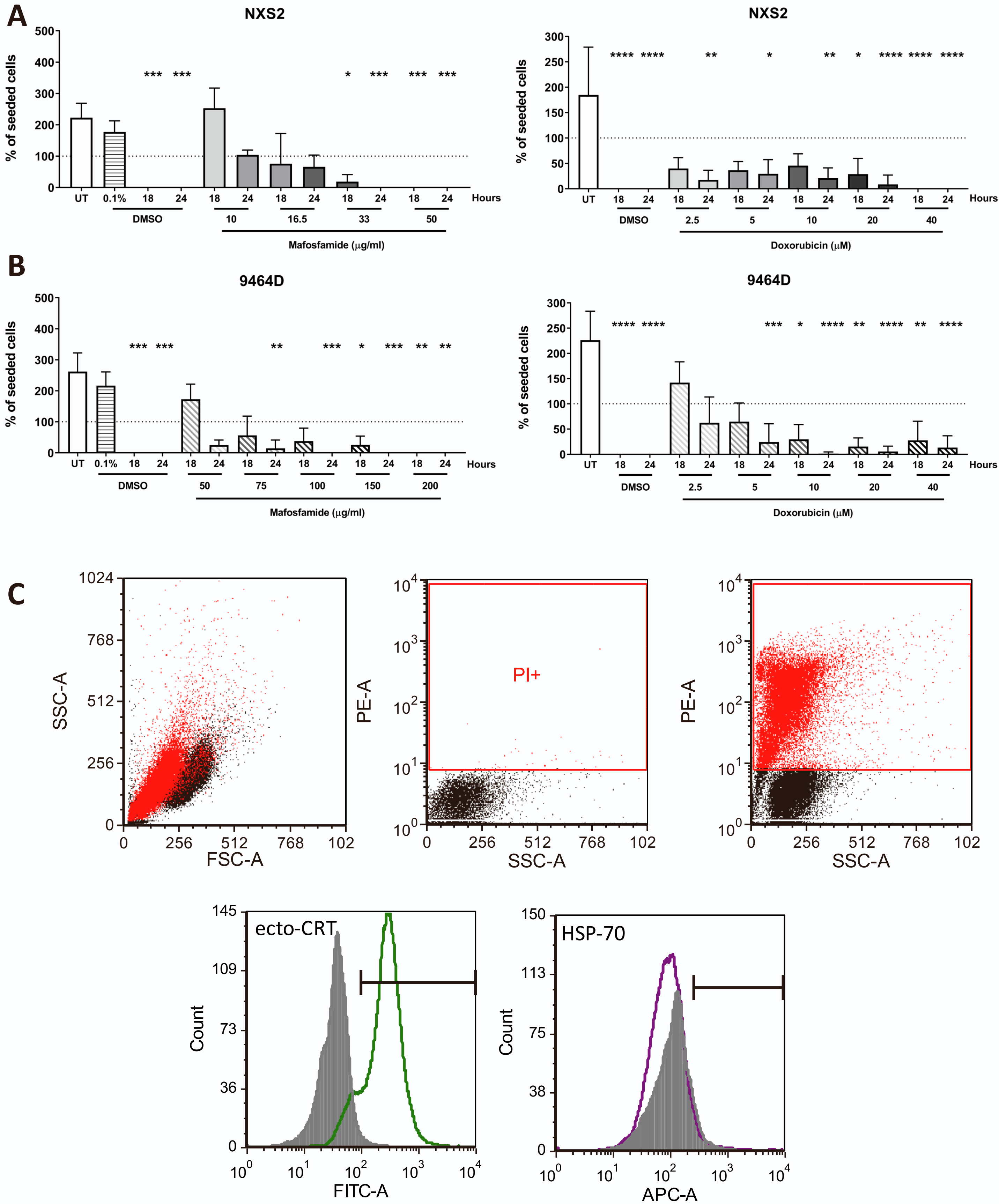

Figure.S1 Murine neuroblastoma cell lines are susceptible to immunogenic cell death induction after chemotherapy application in vitro. Related to Figure 1. A) NXS2 and **B)** 9464D cells were plated at $5x10^4$, either MAF or DOX was added at the doses stated on the figure for 24hrs. Cell proliferation and viability was assessed using an MTT assay. **C)** Cells were plated at $5x10^4$ then treated with either MAF (50 μg/ml NXS2; 75 μg/ml 9464D) or DOX (40 μM) for 24 hrs, then assessed for PI staining, and ecto-CRT and HSP-70 expression by flow cytometry. Shown is example of flow cytometry gating, n=5-6, performed over at least 2 independent experiments. Data are represented as mean +/- SD. Significance was assessed by t-test with * = p <0.05, ** = p <0.01, *** = p <0.001, **** = p<0.0001.

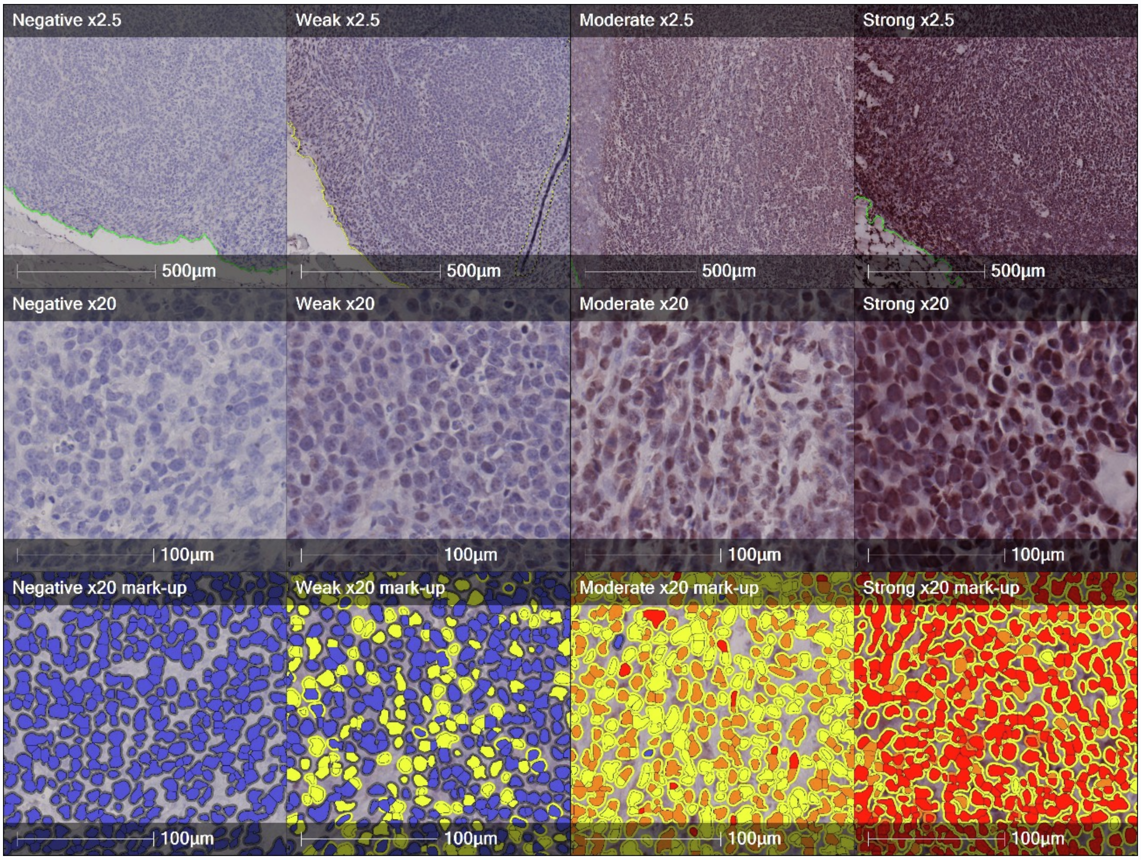

Figure.S2 Quantification of HMGB1 staining on paraffin embedded tumours by IHC. Related to Figure 1. 9464D tumours were formalin fixed and paraffin embedded at Day 3 post CPM (40 mg/kg), and control stained with HMGB1 antibody. 10x magnification. Example shown of how quantification of HMGB1 staining was conducted by pathologist, as detailed in methods.

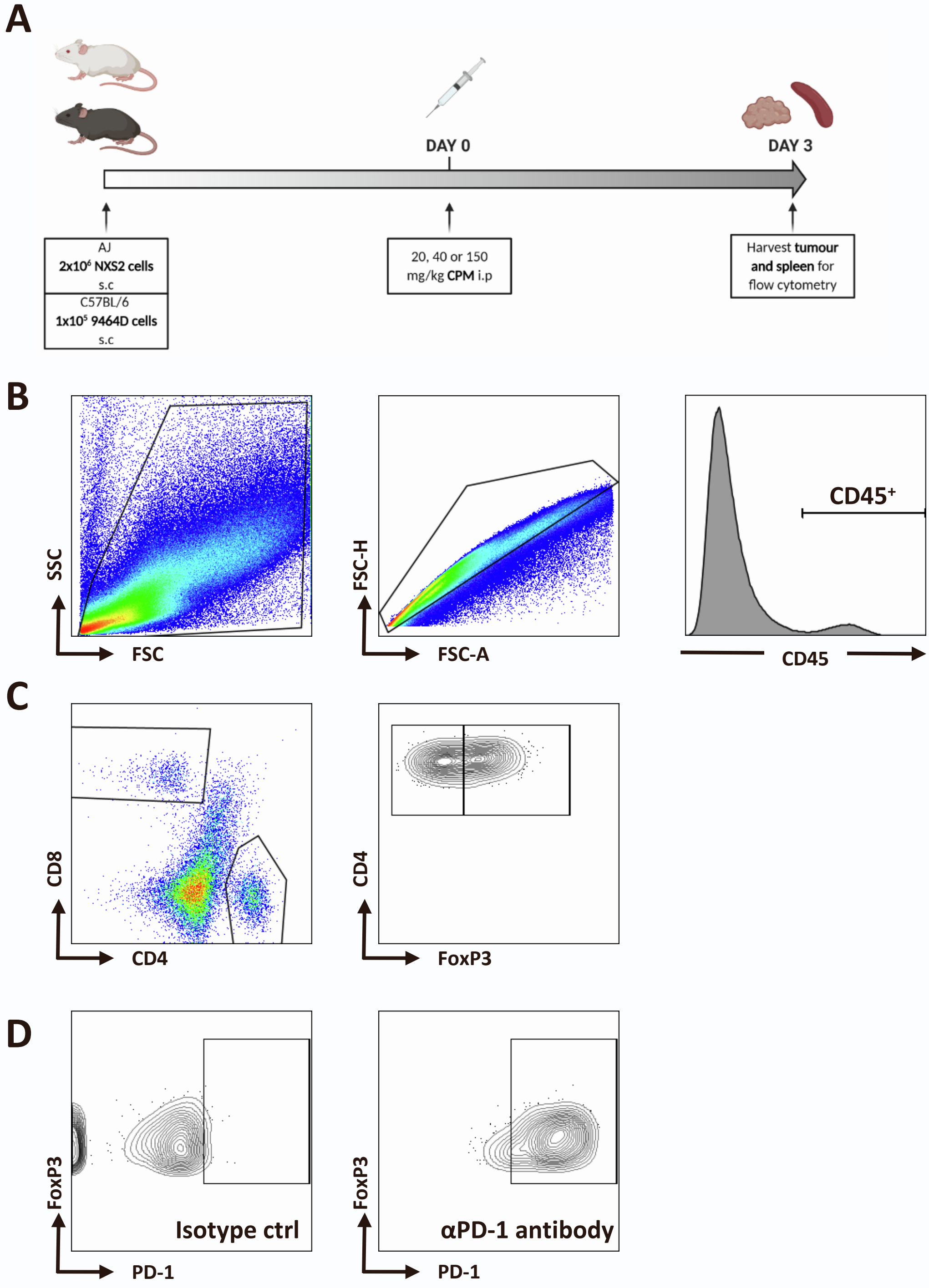

Figure.S3 Gating strategies for tumour, spleen, blood and LN for lymphocyte populations. Related to Figure.2 and Figure.3. A) Schematic example of immunophenotyping of CPM treated tumours. Created in Biorender. **B)** Example of flow cytometry gating for immunophenotyping of tumours. Debris removal, singlet discrimination and CD45+ cell selection is shown. **C)** $CD4^+$ and $CD8^+$ gating, $FoxP3^+$ and $FoxP3^-$ cell gating on $CD4^+$ population. **D)** Example of gating for T cell markers on $FoxP3^+$ cells using PD-1, with isotype control staining shown on left and anti-PD-1 antibody staining shown on right.

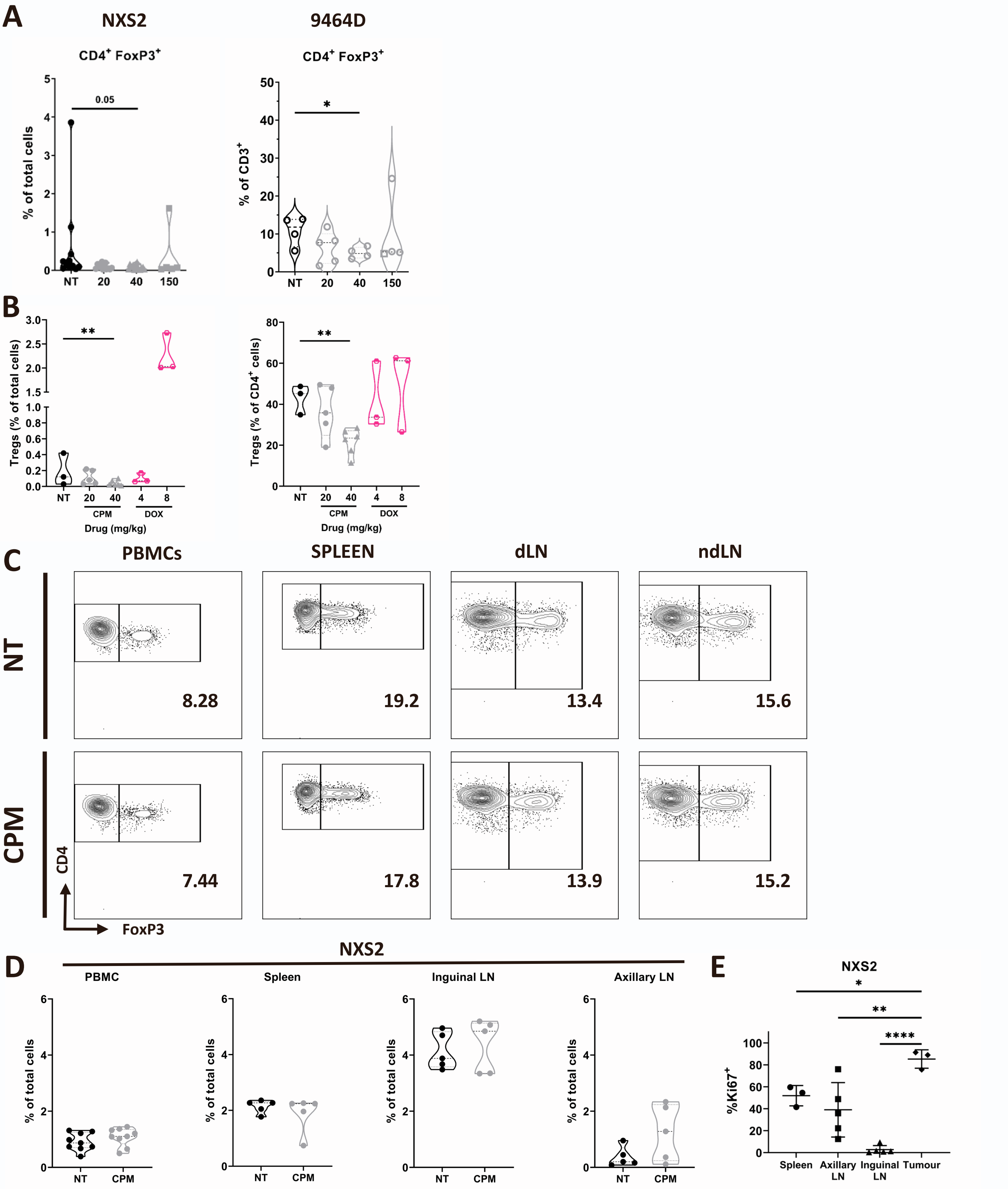

Figure.S4 Further immunophenotyping data of CPM treated tumours. Related to Figure.2. A) Treg (CD4+ FoxP3+) percentage in NXS2 tumours shown as a percentage of total cells (left) or in 9565D tumours as a percentage of CD3+. n=5-11, combination of two separate experiments. **B)** NXS2 tumour bearing A/J mice were treated with either CPM or DOX, with tumours harvested at Day 3 for immunophenotyping with Tregs (CD4+ FoxP3+) shown as percentage of total cells (left) or CD4+ (right). n=3-6, performed over 2 independent experiments. **C)** Example of flow cytometry gating of FoxP3+ and FoxP3- cells on CD4+ populations in NT and CPM treated PBMCs, spleens, draining lymph nodes (dLN) and non-draining lymph nodes (ndLN), with percentage of FoxP3+ noted. **D)** Percentage of CD4+ FoxP3+ cells as % of total cells for NXS2 tumour bearing mice. N=9 (PBMC) or 5 (Spleen and LNs). **E)** Percentage Ki67+ cells shown as a percentage of FoxP3+ cells in numerous tissues of NXS2 tumour bearing mice. N=3 (spleen+ tumour); n= 5 (Axillary and inguinal LN). Data are represented as mean +/- SD. Significance was assessed by t-test with * = p <0.05, ** = p<0.01, *** = p<0.001 and **** = p<0.0001.

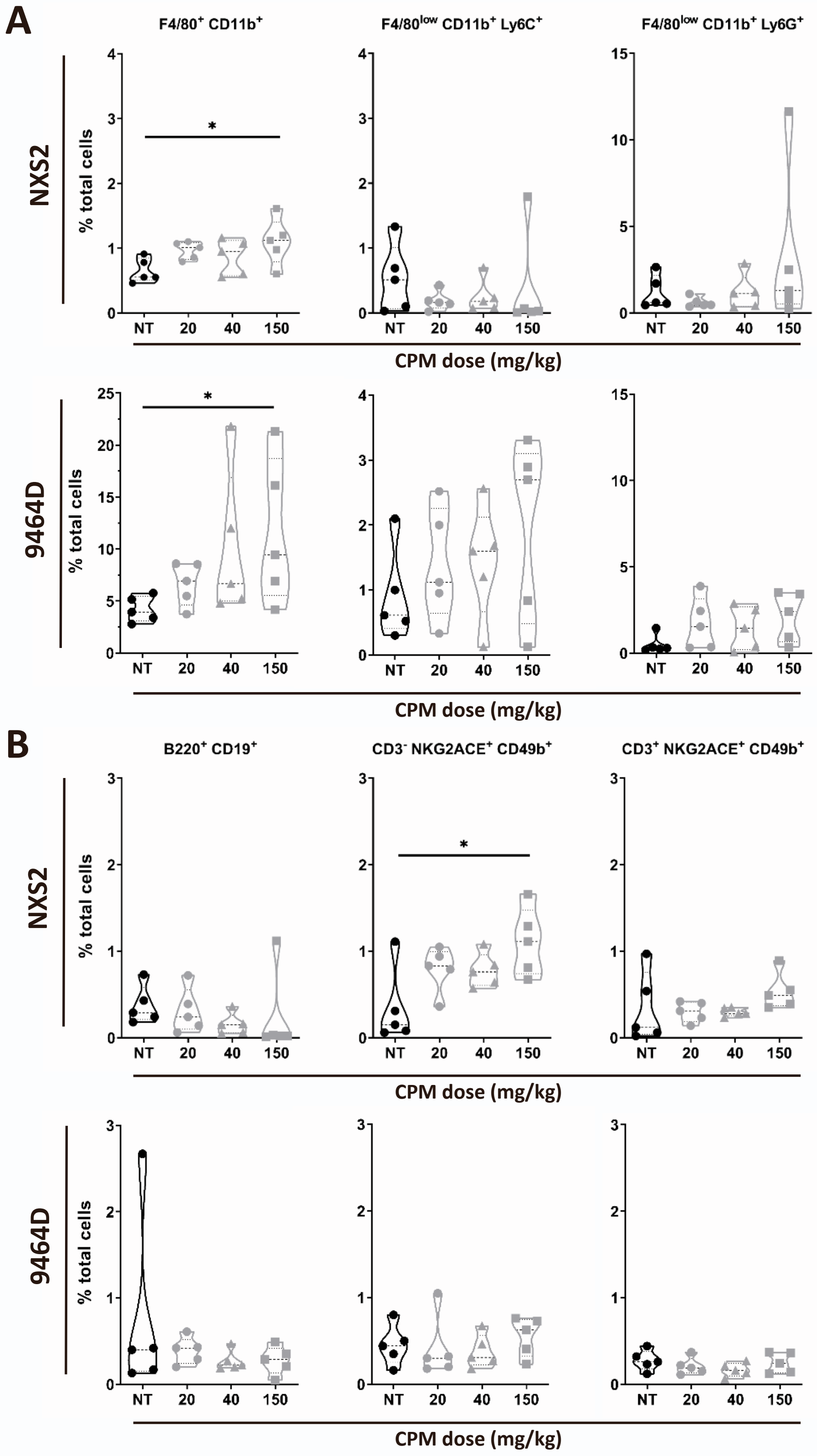

Figure.S5 Low dose CPM does not significantly modulate myeloid, NK and B cell populations. Related to Figure.2. A) A/J or C57BL/6 mice were subcutaneously injected with NXS2 or 9464D cells respectively. At tumour size of 8x8 mm, mice were injected i.p with the corresponding dose of CPM. After 3 days, mice were culled and tissues were harvested for immunophenotyping. Percentage of tumour infiltrating macrophages (F4/80$^+$ CD11b$^+$), monocytes (F4/80^{low} CD11b$^+$ Ly6C$^+$) and neutrophils (F4/80^{low} CD11b$^+$ Ly6G$^+$) and **(B)** B cells (B220$^+$ CD19$^+$), NK cells (CD3$^-$ NKG2ACE$^+$ CD49b$^+$) and NK T cells (CD3$^+$ NKG2ACE$^+$ CD49b$^+$) was determined by flow cytometry, using the gating shown in Fig.S3. n = 5 mice per group, performed over at least 2 experiments. Data are represented as mean +/- SD. Significance was assessed by t-test with * = $p < 0.05$ and ** = $p < 0.01$.

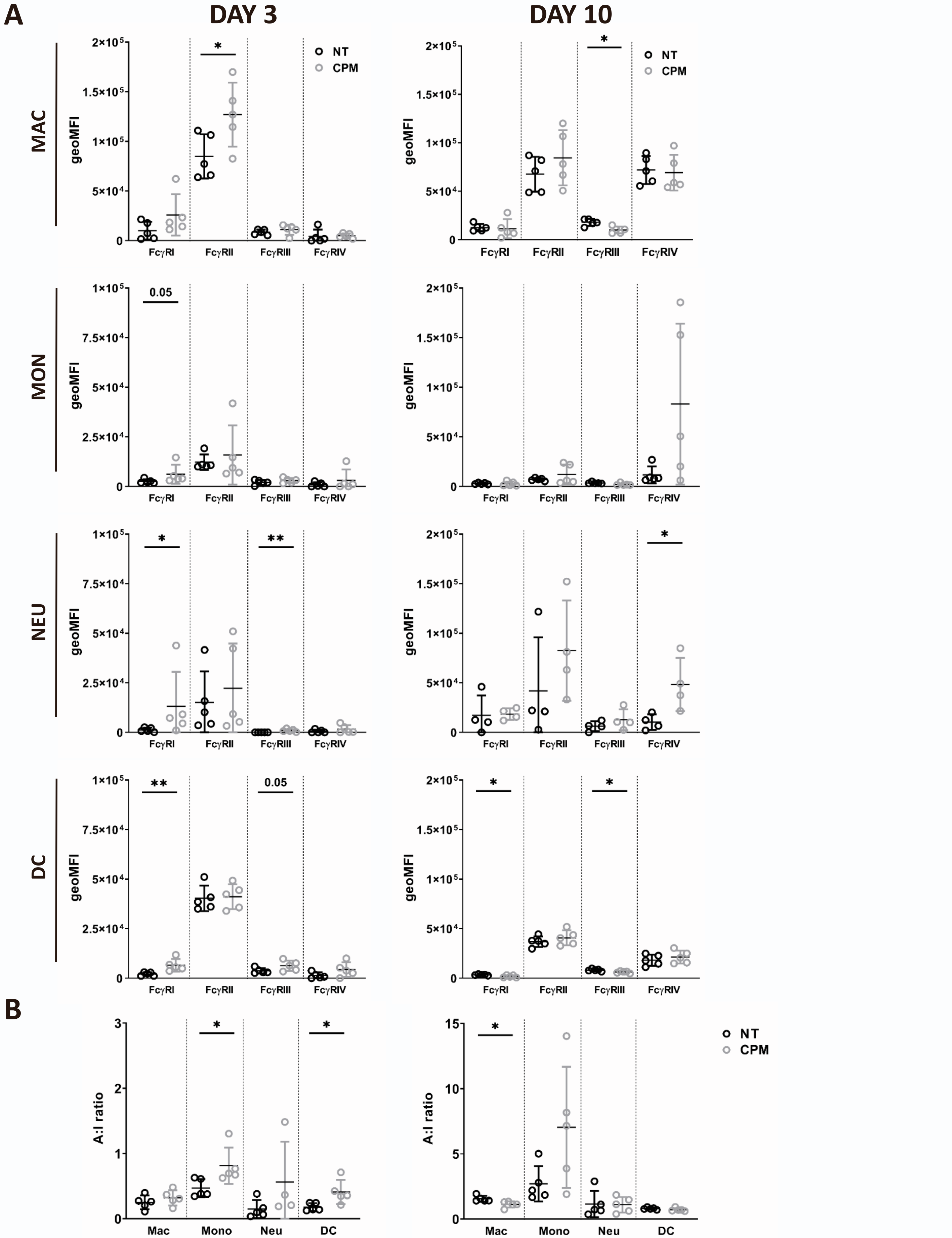

Figure.S6 FcγR expression on myeloid cells post CPM administration. Related to Figure.2 A) C57BL/6 mice were subcutaneously injected with 9464D cells. At tumour size of 8x8 mm, mice were injected i.p with the 40 mg/kg of CPM. After Day 3 or Day 10, mice were culled and tissues were harvested for immunophenotyping by flow cytometry. Macrophage, monocyte and neutrophil populations were gated as demonstrated in Fig.S3. Analysis of FcγR expression on each population was measured and shown as geoMFI. **B)** Activatory to inhibitory (A:I) ratio of FcγR. n = 4-5 mice per group performed in one experiment. Data are represented as mean +/- SD. Significance was assessed by t-test with * = $p < 0.05$ and ** = $p < 0.001$.

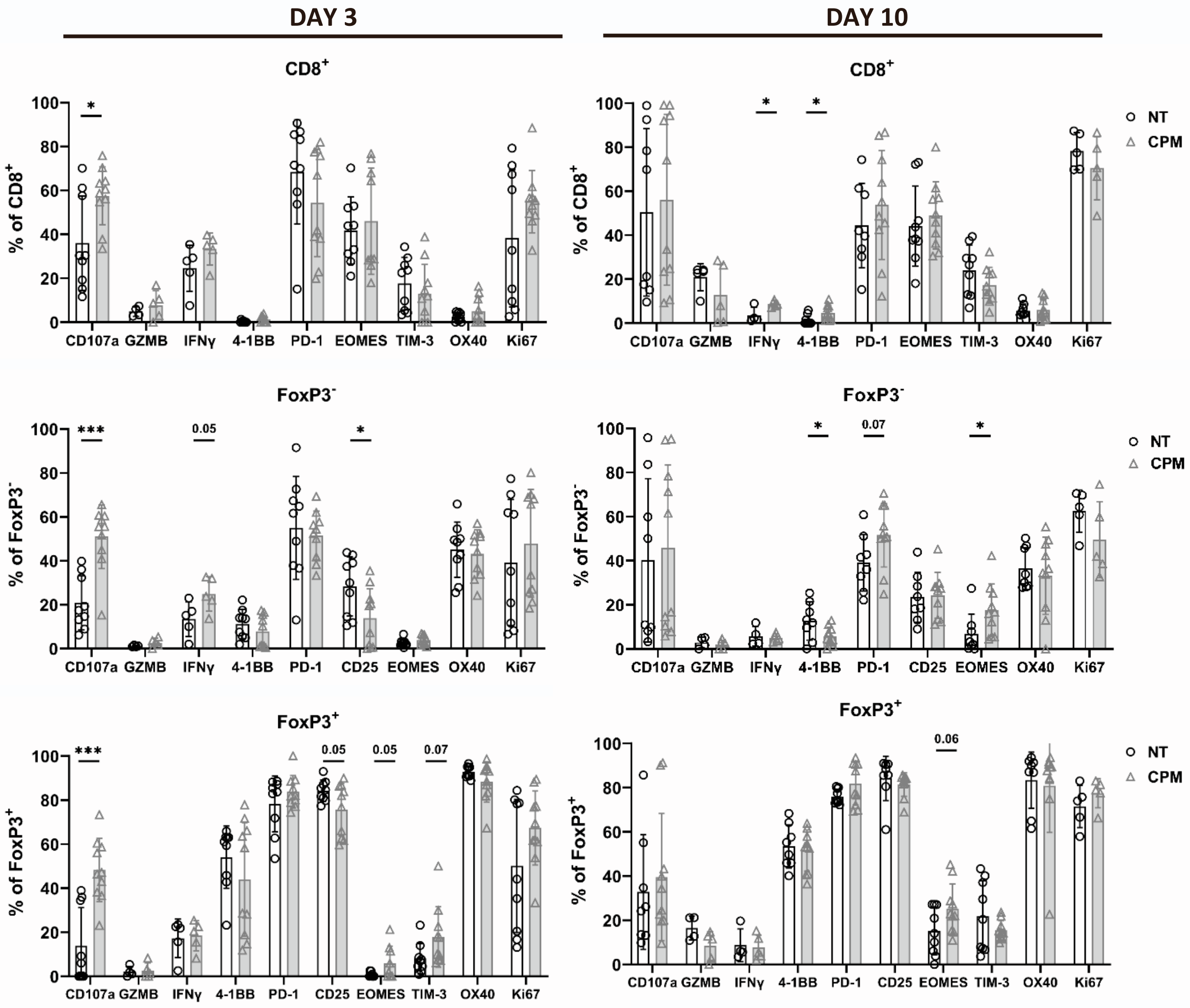

Figure.S7 Percentage positive of T cell subset immunophenotyping. Related to Figure.3. Mice bearing 9464D tumours were injected with 40 mg/kg CPM i.p once tumours reached 8x8 mm. Mice were culled and tissues harvested at either Day 3 or Day 10 post CPM, for immunophenotyping analysis, using flow cytometry. CD8+, CD4+ FoxP3- and CD4+ FoxP3+ populations were gated as shown in Fig.S3. Percentage expression of several T cell proteins was assessed at both Day 3 and Day 10. n= 4-10, performed over 2 experiments. Data are represented as mean +/- SD. Significance was assessed by t-test with * = p <0.05, ** = p <0.01, *** = p <0.001, **** = p<0.0001.

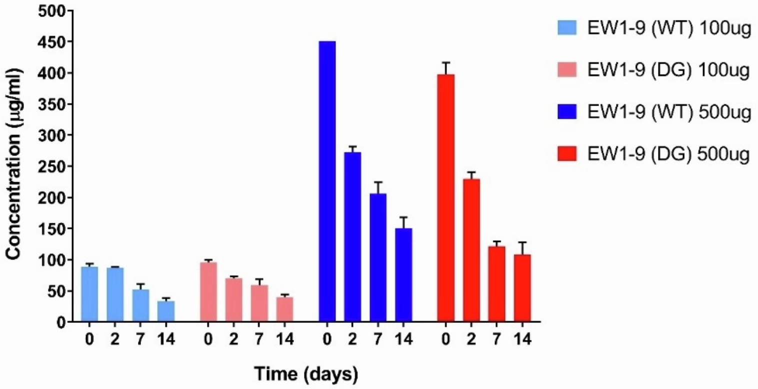

Figure.S8 Deglycosylation of EW1-9 antibody does not affect circulating concentration levels in vivo. Related to Figure 5. Wild type (WT) anti-PD-1 (Clone: EW1-9) was deglycosylated (DG) using PNGase F. C57BL/6 mice were injected with either 100 µg or 500 µg of EW1-9 (WT or DG) i.p. Serum samples were analysed for antibody concentration using ELISA. Data are represented as mean +/- SD. n=3 per group performed in one experiment.

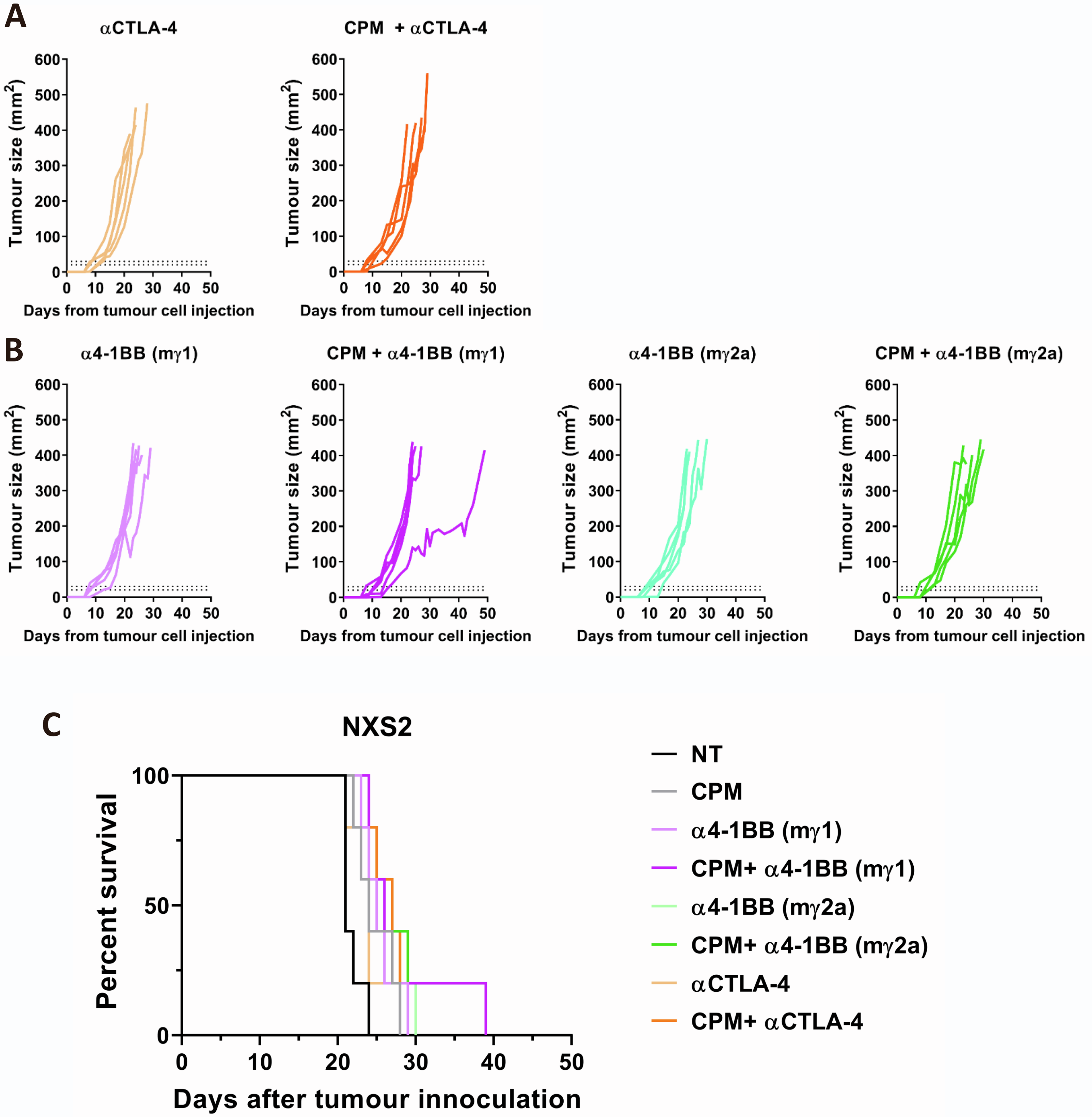

Figure.S9 Combination therapy with either anti-4-1BB or anti-CTLA-4 antibodies do not significantly increase survival. Related to Figure.5. A) A/J mice were injected with NXS2 cells subcutaneously. Once tumours reached 8x8 mm (Day 0) mice were randomised into groups and treated with either CPM (40 mg/kg) or PBS. At Day 3 mice were treated i.p with 250 µg of antibody as described in figures, followed by a second dose at Day 6. Tumour growth was monitored and survival recorded at end point. Tumour growth for αCTLA-4 and CPM +αCTLA-4 groups. **B)** Tumour growth for α4-1BB (mouse IgG1 = mγ1), CPM +α4-1BB (mγ1), α4-1BB (mouse IgG2a = mγ2a) and CPM +α4-1BB (mγ2a). **C)** Survival shown for all groups. Survival data for NT and CPM used for Figure 1A. n= 5 per group, performed in one experiment. Significance assessed by Log-Rank test (D).

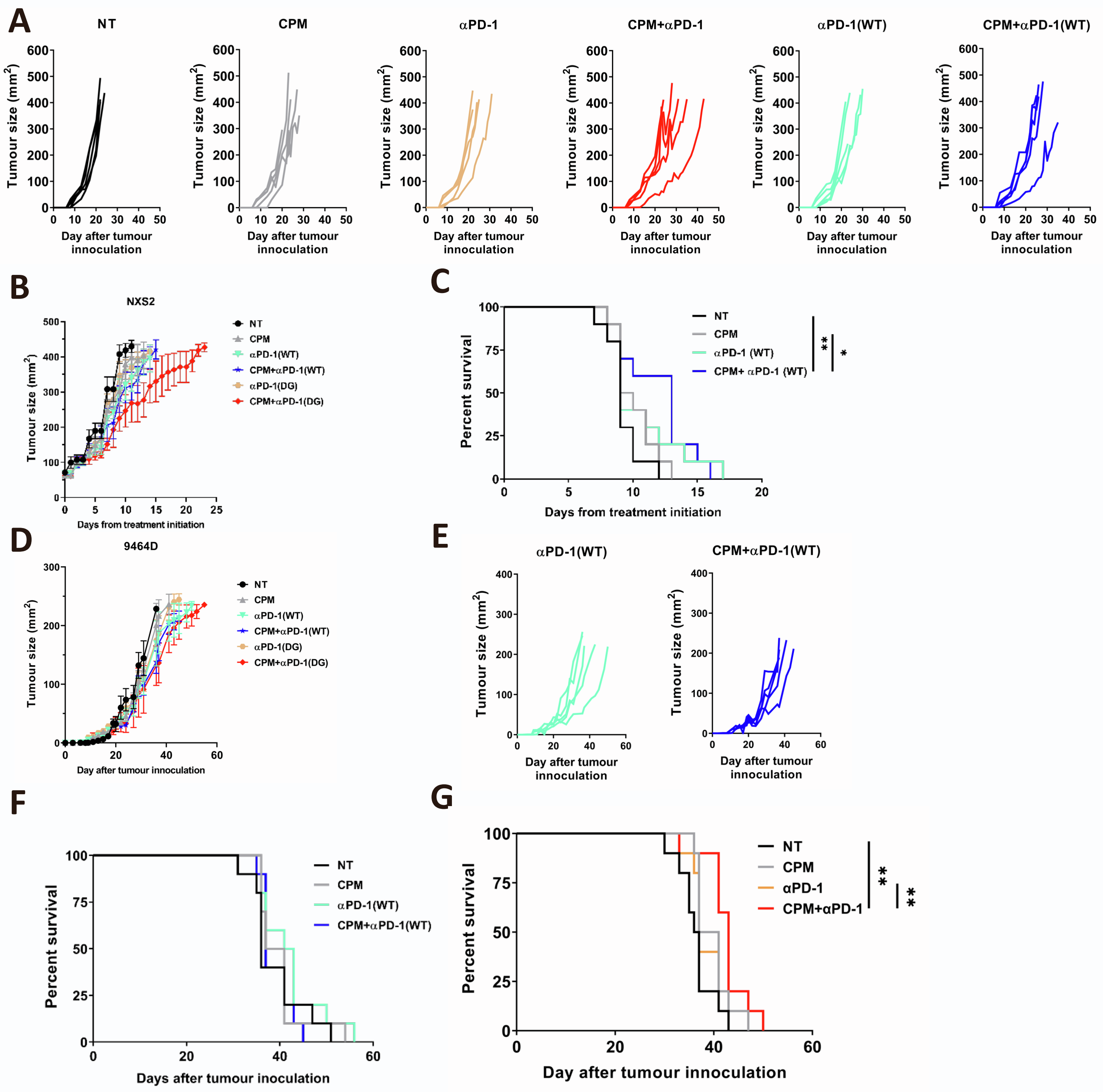

Figure.S10 Tumour growth and survival after anti-PD-1 WT combination with CPM treatment. Related to Figure 5. A) A/J mice were injected with NXS2 cells subcutaneously. Once tumours reached 8x8 mm (Day 0) mice were treated with either CPM (40 mg/kg) or PBS. At Day 3 mice were treated i.p with 250 µg of either αPD-1 (WT), αPD-1 (DG) or PBS as described in figures, followed by a second dose at Day 6. Tumour growth was monitored with tumour growth shown for all groups. **B)** Average tumour growth for all groups. **C)** Survival shown for NT, CPM, αPD-1 (WT) and CPM+ αPD-1 (WT) groups. **D)** 9464D mice were injected subcutaneously with 9464D cells. At palpable tumour size (Day 0) mice were treated as described in A. Average tumour growth shown **E)**. Individual mice tumour growth is shown for αPD-1 (WT) and CPM+ αPD-1 (WT) groups, with survival shown in **F)**. **G)** Survival analysis of repeat experiment of Fig.5E-G at larger tumour start point of 5x5 mm. n=5-10 (A-G), example shown of two experiments. n=8-10 (H), combination of two separate experiments. Data are represented as mean +/- SD. Significance was assessed by Log-Rank test with * = p<0.05 and ** = p<0.01.
